# Triple Play of Band Gap, Interband, and Plasmonic Excitations for Enhanced Catalytic Activity in Pd/H_*x*_MoO_3_ Nanoparticles in the Visible Region

**DOI:** 10.1021/acsami.3c17101

**Published:** 2024-02-21

**Authors:** Leticia S. Bezerra, Samir A. Belhout, Shiqi Wang, Jhon Quiroz, Paulo F.M. de Oliveira, Shwetha Shetty, Guilherme Rocha, Hugo L. S. Santos, Sana Frindy, Freddy E. Oropeza, Víctor A. de la Peña O’Shea, Antti-Jussi Kallio, Simo Huotari, Wenyi Huo, Pedro H.C. Camargo

**Affiliations:** † Department of Chemistry, University of Helsinki, A.I. Virtasen aukio 1, PO Box 55, Helsinki 00014, Finland; ‡ Departamento de Química Fundamental, Instituto de Química, Universidade de São Paulo. Av. Lineu Prestes 748, São Paulo 05508000, Brazil; § Photoactivated Processes Unit, IMDEA Energy Institute, Avda. Ramón de la Sagra 3, Mostoles, Madrid 28935, Spain; ∥ Department of Physics, University of Helsinki, P.O. Box 64, Helsinki 00014, Finland; ⊥ College of Mechanical and Electrical Engineering, Nanjing Forestry University, Nanjing 210037, P. R. China; # NOMATEN Centre of Excellence, National Centre for Nuclear Research. Otwock 05-400, Poland

**Keywords:** plasmonic photocatalysis, earth-abundant materials, mechanochemical synthesis, hydrogen evolution reaction, phenylacetylene hydrogenation

## Abstract

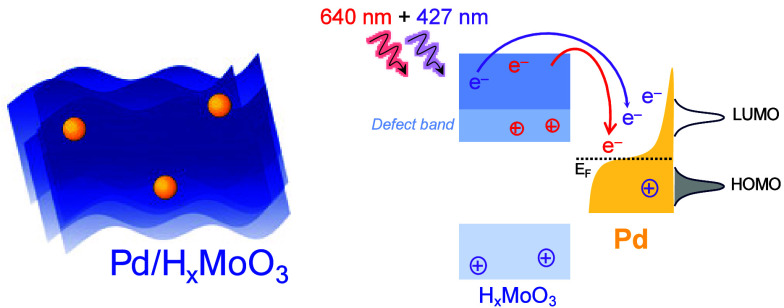

Plasmonic photocatalysis has been limited by the high cost and scalability of plasmonic materials, such as Ag and Au. By focusing on earth-abundant photocatalyst/plasmonic materials (H_*x*_MoO_3_) and Pd as a catalyst, we addressed these challenges by developing a solventless mechanochemical synthesis of Pd/H_*x*_MoO_3_ and optimizing photocatalytic activities in the visible range. We investigated the effect of H_*x*_MoO_3_ band gap excitation (at 427 nm), Pd interband transitions (at 427 nm), and H_*x*_MoO_3_ localized surface plasmon resonance (LSPR) excitation (at 640 nm) over photocatalytic activities toward the hydrogen evolution and phenylacetylene hydrogenation as model reactions. Although both excitation wavelengths led to comparable photoenhancements, a 110% increase was achieved under dual excitation conditions (427 + 640 nm). This was assigned to a synergistic effect of optical excitations that optimized the generation of energetic electrons at the catalytic sites. These results are important for the development of visible-light photocatalysts based on earth-abundant components.

## Introduction

Visible-light photocatalysis is a crucial technology to enable solar-driven chemistry and be the stepping stone for the green transition of our society.^1,2^ Photocatalysis can address important molecular transformations related to energy generation, synthesis of commodity chemicals, providing a clean environment, mitigating climate change, and enabling a circular economy.^3,4^ Nevertheless, photocatalytic performances in the visible region are hindered by the fact that most employed photocatalytic systems based on semiconductors require UV light for activation, limiting their efficiency for solar-driven chemistry.^5,6^

In this context, recent advances in plasmonic catalysis based on the localized surface plasmon resonance (LSPR) excitation in plasmonic nanoparticles (NPs) have enabled high photocatalytic enhancements in the visible range for a variety of transformations and catalyst designs.^7−10^ They comprise, for example, plasmonic NPs having both LSPR and catalytic properties; antenna–reactor NP design coupling a plasmonic (antenna) and catalytic (reactor) component in the form of alloy; and core–shell, core–satellite, and hybrid materials composed of a combination of plasmonic and semiconductor materials.^11−14^ Despite their potential, one of the bottlenecks is that most common plasmonic materials are composed of expensive metals such as Ag and Au NPs. Although alternative earth-abundant materials have been proposed, such as Al, Cu, metal nitrides, and metal oxides,^15−20^ these systems may remain limited in terms of performance and large scalability of their synthesis. Therefore, the development of high-performing visible light photocatalysts that are based on abundant elements and that can be prepared by scalable and environmentally friendly processes is the piece of the puzzle needed to enable photocatalysis to reach its full potential.

In this paper, we address these challenges by focusing on H_*x*_MoO_3_ as an earth-abundant photocatalytic and plasmonic semiconducting component (antenna) coupled with Pd NPs as the catalytic sites (reactor) in a model hybrid NP design. They are composed of Pd NPs supported onto defective H_*x*_MoO_3_, named Pd/H_*x*_MoO_3_. First, we prepared this material in one pot at room temperature and under solventless conditions by a mechanochemical approach followed by H-spillover. Then, we present a comprehensive investigation and optimization of photocatalytic properties in the visible region through the control of different optical excitation processes via distinct light illumination conditions. As H_*x*_MoO_3_ supports band gap and LSPR excitation whereas Pd supports interband transitions (all in the visible range), this unique combination of excitation processes allows us to explore and understand how different light wavelengths impact the catalytic activity of Pd sites in the presence of H_*x*_MoO_3_. Here, we employed 427 nm to probe the effect of the H_*x*_MoO_3_ band gap excitation and Pd interband transitions on catalytic activities, whereas 640 nm could be employed to focus on the impact of H_*x*_MoO_3_ LSPR excitation. By investigating the hydrogen evolution reaction (HER) and phenylacetylene hydrogenation reaction as model transformations, we have found that both 427 and 640 nm excitation led to comparable enhancements in catalytic activity for both transformations. Intriguingly, we found that the catalytic activity was significantly boosted, achieving a 110% photoenhancement, when we employed dual excitation conditions (both 427 and 640 nm simultaneously). This could be explained by the synergistic effect of the H_*x*_MoO_3_ band gap, H_*x*_MoO_3_ LSPR, and Pd interband excitation, which collectively optimized the generation of energetic electrons at the catalytic sites (Pd) under light illumination. Therefore, these results provide valuable insights into the design and development of high-performance photocatalysts and the optimization of photocatalytic properties.

## Results and Discussion

**Scheme 1:**
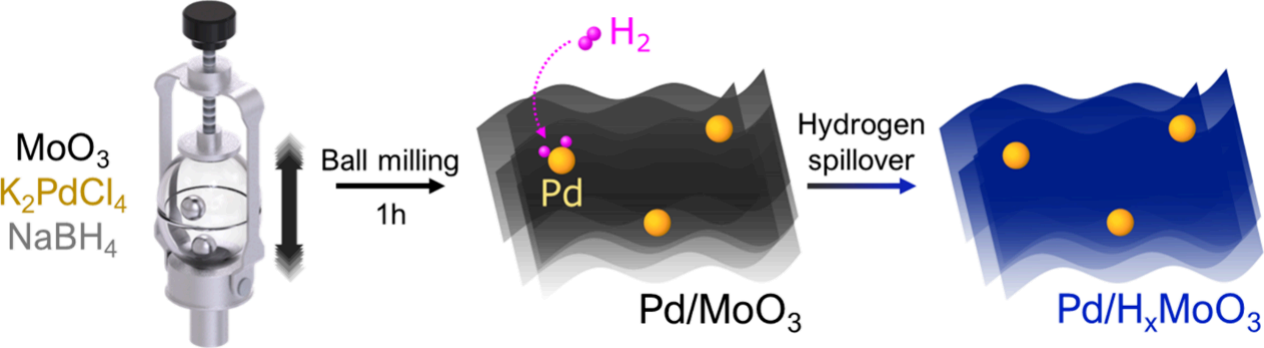
Synthesis of Pd/H_*x*_MoO_3_ Plasmonic Photocatalysts Scheme for the mechanochemical approach to produce Pd/MoO_3_ followed by Pd-assisted hydrogen spillover to yield Pd/H_*x*_MoO_3_. This method employed commercial MoO_3_ and K_2_PdCl_4_ as starting materials and NaBH_4_ as a reducing agent

The synthesis of Pd NPs supported on MoO_3_ was performed via a mechanochemical process as described in Scheme 1. In this process, the Pd precursor was reduced by the NaBH_4_ leading to the deposition of Pd NPs on MoO_3_ while the MoO_3_ structure was exfoliated (due to the mechanochemical forces).^21^ This leads to the formation of Pd/MoO_3_. During this process, H^–^ species generated from NaBH_4_ can also lead to some degree of H-intercalation onto the MoO_3_ structure and oxygen removal.^21^ To endow Pd/MoO_3_ with plasmonic properties (support LSPR in the visible region), hydrogen intercalation into the MoO_3_ structure was performed via hydrogen spillover by bubbling H_2_ gas into a suspension containing Pd/MoO_3_, leading to the formation of Pd/H_*x*_MoO_3_. In this process, H_2_ molecules undergo dissociative chemisorption on the Pd surface, forming adsorbed H atoms that can easily migrate from the Pd surface to the MoO_3_ lattice and produce a dark blue material known as hydrogen molybdenum bronze (H_*x*_MoO_3_).^22−24^ The intercalation of H-species leads to an increase in the presence of free charge carriers a with a high degree of delocalization, leading to LSPR in H_*x*_MoO_3_.^22,23^ As H_*x*_MoO_3_ is also a semiconductor, in terms of photocatalysis applications, H_*x*_MoO_3_ can support both band gap (semiconductor photocatalysis) and LSPR excitations (plasmonic catalysis) at different wavelengths.^25,26^ Moreover, it is established that Pd NPs can undergo interband excitations at wavelengths similar to the band gap excitation of H_*x*_MoO_3_.^27,28^ This makes this system, Pd/H_*x*_MoO_3_, ideal for investigating the effect of the following transitions to optimize catalytic activities under visible light excitation: (1) the combined effect of H_*x*_MoO_3_ band gap excitation and Pd interband transitions; (2) the effect of the LSPR excitation of H_*x*_MoO_3_; and (3) the triple play of H_*x*_MoO_3_ band gap excitation, Pd interband transitions, and H_*x*_MoO_3_ LSPR excitation. These are the goals that this paper aims to address.

**Figure 1:**
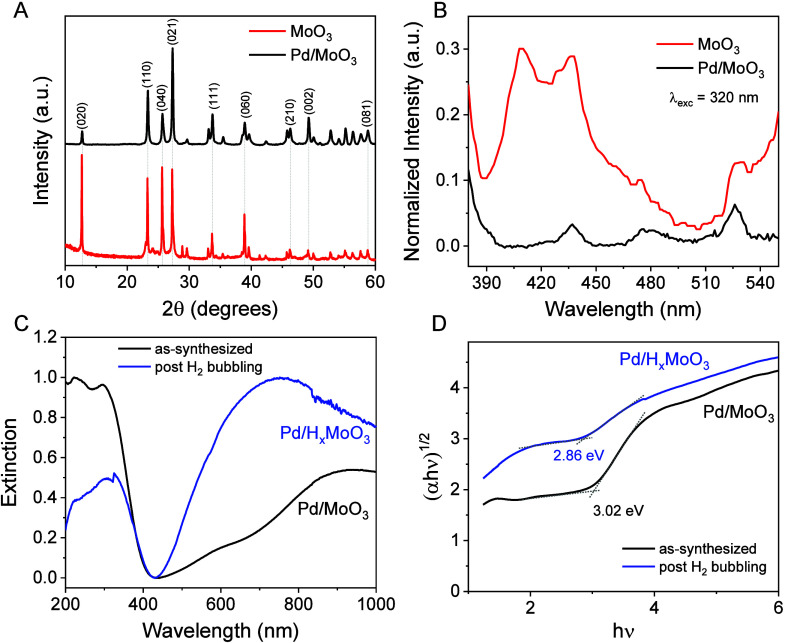
Characterization of Pd/MoO_3_ and Pd/H_*x*_MoO_3_ samples. (A) Powder XRD patterns and (B) photoluminescence spectra for the Pd/MoO_3_ and MoO_3_ materials. (C) UV–vis extinction spectra and (D) corresponding Tauc plots for Pd/MoO_3_ and Pd/H_*x*_MoO_3_ (before and after H_2_ bubbling) dispersed in isopropanol. MoO_3_, Pd/MoO_3_, and Pd/H_*x*_MoO_3_ are denoted by red, black, and blue traces, respectively.

The XRD patterns for the MoO_3_ and Pd/MoO_3_ materials are presented in Figure 1A. The diffraction peaks at 2θ: 12.7, 23.3, 25.7, 27.3, 33.8, 35.5, 38.9, 46.3, 49.2, and 58.8° were indexed to the (002), (110), (040), (021), (111), (041), (060), (210), (002), and (081) planes of orthorhombic MoO_3_ (α-MoO_3_) (JCPDS 005-0508).^29^ For Pd/MoO_3_, no peaks from Pd were observed because of the low Pd content in the sample (0.83 wt % as measured by AES). The minor reflections close to (110) and (040) detected for Pd/MoO_3_ are assigned to the formation of the HxMO_3_ structure, which is partially formed during the mechanochemical step.^21^ This is further illustrated by the XRD patterns obtained for Pd/H_*x*_MO_3_ after the H_2_ bubbling step (Figure S1), which shows XRD peaks assigned to both MoO_3_ and H_*x*_MO_3_ phases. The crystallite sizes calculated by using the Scherrer equation corresponded to 43.1 and 35.8 nm for MoO_3_ and Pd/MoO_3_, respectively. The decrease in crystallite size agrees with the mechanochemical synthesis that led to the exfoliation of the MoO_3_ structure.^21^Figure 1B shows the room temperature photoluminescence spectra of MoO_3_ and Pd/MoO_3_ obtained with a 320 nm excitation wavelength. The main emission bands for MoO_3_ located at 409 and 436 nm are attributed to the radiative electron–hole recombination between conduction and valence band.^30−32^ The lower energy bands located at 475 and 526 nm are related to electron–hole recombination between conduction band and defect sublevels created by surface defects or oxygen vacancies.^30,32^ For the Pd/MoO_3_ spectrum, the signal intensities decreased, suggesting a strong interaction between the support MoO_3_ and Pd NPs in which the presence of Pd improves the efficiency of electron–hole separation and suppresses the recombination of electron–hole pairs.

The Mo K-edge XANES spectra of as-synthesized MoO_3_ and Pd/MoO_3_ samples are shown in Figure S2, along with the Mo foil and commercial MoO_3_ reference spectra. The commercial MoO_3_ exhibited a discrete pre-edge peak at 20.005 keV followed by edge absorption at 20.02 keV, and both MoO_3_ and Pd/MoO_3_ exhibited similar features. The pre-edge feature is related to the quadrupole-allowed transitions from 1s to 4d levels, and the edge feature is related to the dipole-allowed transitions from 1s to 5p levels. Furthermore, the edge energy is indicative of an oxidation state, which is evidenced by the Mo foil spectra with a lower edge absorption (20.01 keV) compared to MoO_3_ samples. The shift in absorption edges to higher energies indicates a higher oxidation state.^33,34^

To investigate the effect of H_2_ bubbling and thus hydrogen spillover, the UV–vis absorption spectra of Pd/MoO_3_ and Pd/H_*x*_MoO_3_ (before and after H_2_ bubbling, respectively) are shown in Figure 1C. The as-synthesized Pd/MoO_3_ showed a strong absorption band in the UV region due to band gap excitation typical of semiconductors,^35^ with an absorption edge around 425 nm and discrete absorption in the visible and near-IR regions, which may be associated with a low degree of hydrogen doping in the MoO_3_ structure during the synthesis with NaBH_4_. After the H_2_ bubbling, the solution changed color from gray to dark blue accompanied by an increase in the LSPR peak from 450 to 900 nm, characteristic of plasmonic H_*x*_MoO_3_. The intercalation of H atoms into the van der Waals gap of MoO_3_ induces intrinsic defects and injects electrons into the framework, changing the electronic structure, band gap, and conductivity.^22,29,36^ The optical band gaps of Pd/MoO_3_ before and after H_2_ bubbling were also determined from the Tauc plots in Figure 1D and showed a slight decrease from 3.02 to 2.86 eV (433 nm) after the H intercalation, in agreement with the reported band gap for plasmonic MoO_3_.^37−39^ These results showed that the H_2_ bubbling step efficiently induces plasmonic properties into the MoO_3_ support via the generation of H_*x*_MoO_3_ due to hydrogen spillover, which is also supported by XRD data (Figure S1).^36,40,41^ In this process, H_2_ interacts with the Pd surface, dissociates, and migrates into the MoO_3_ lattice. This occurs through H atom adsorption onto oxygen, causing a charge transfer between the H 1s orbital and the O 2p orbital. In sequence, the O coordinates directly to Mo atoms transferring the extra charge, and the Mo atoms are slightly reduced.^36,40,41^ It is important to note that H_*x*_MoO_3_ can be reversibly transformed to MoO_3_ by gradual oxidation by O_2_ in air under ambient conditions (in the period of 12–48 h).^15,42^

**Figure 2:**
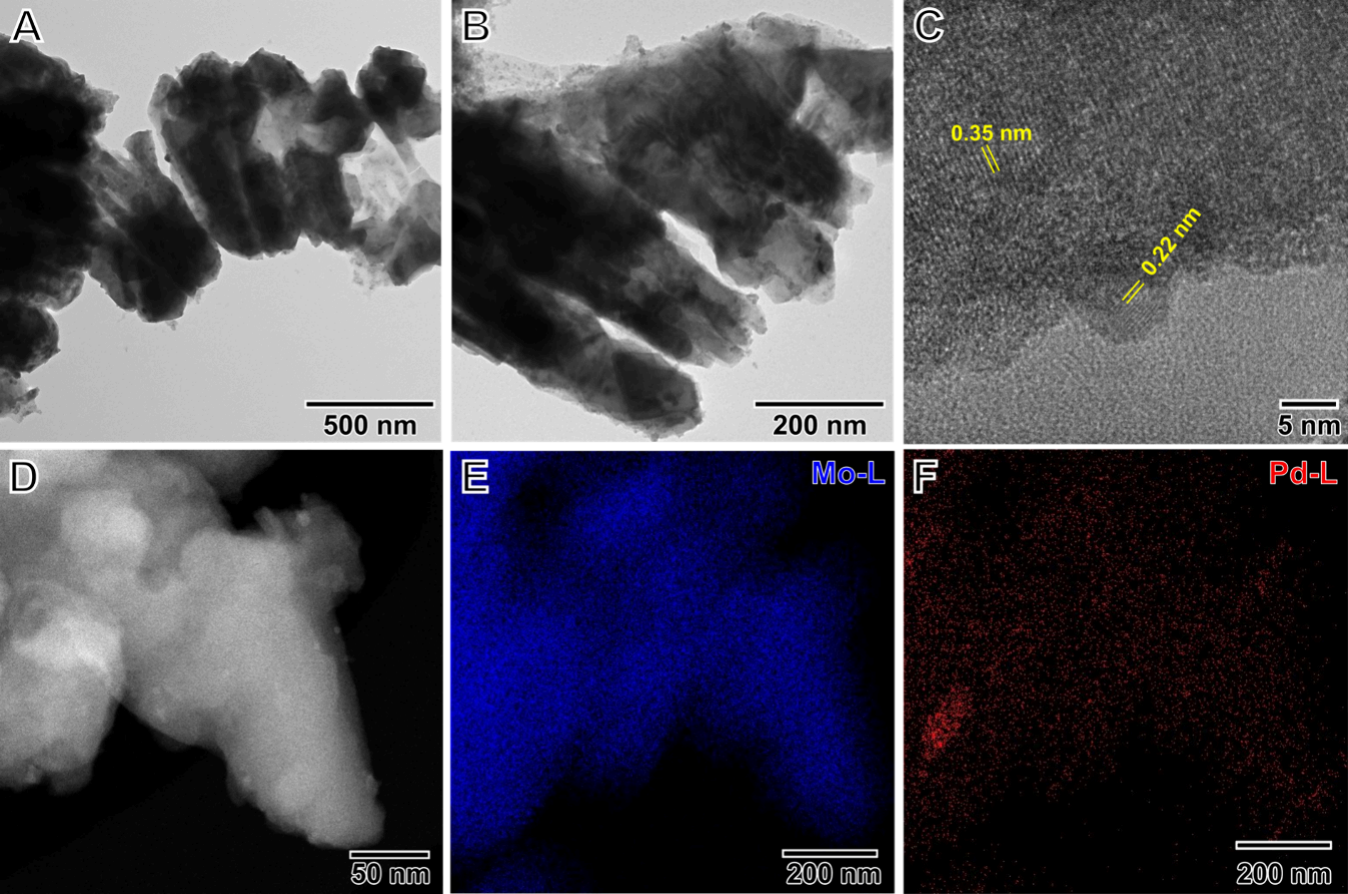
Electron microscopy characterization for Pd/H_*x*_MoO_3_ NPs. (A, B) TEM, (C) HRTEM, and (D) STEM-HAADF images for the Pd/H_*x*_MoO_3_ NPs. (E, F) STEM-EDX mapping for (E) Mo and (F) Pd of the region shown in panel D. The distributions of Mo and Pd are shown in blue and red, respectively.

Figure 2A,B shows transmission electron microscopy (TEM) images for the synthesized Pd/H_*x*_MoO_3_. It reveals that the H_*x*_MoO_3_ was irregular in shape and that the supported Pd NPs were spherical, below 10 nm in diameter, and not uniformly distributed along the sample. This can probably be assigned to the incomplete reduction of the Pd precursors during the mechanochemical process (which was confirmed by the XPS data as discussed below). The HRTEM images (Figure 2C) showed apparent lattice spacings of 0.22 nm corresponding to the (111) plane of Pd structure (JCPDS no. 46-104) and 0.35 nm assigned to the (040) plane of MoO_3_ (ICSD 158256).^43,44^ The STEM-HAADF image (Figure 2D) revealed the presence of Pd NPs over the H_*x*_MoO_3_ support, and STEM-EDX mapping showed that regions containing Mo and Pd were uniformly distributed throughout the sample (Figure 2E,F).

**Figure 3:**
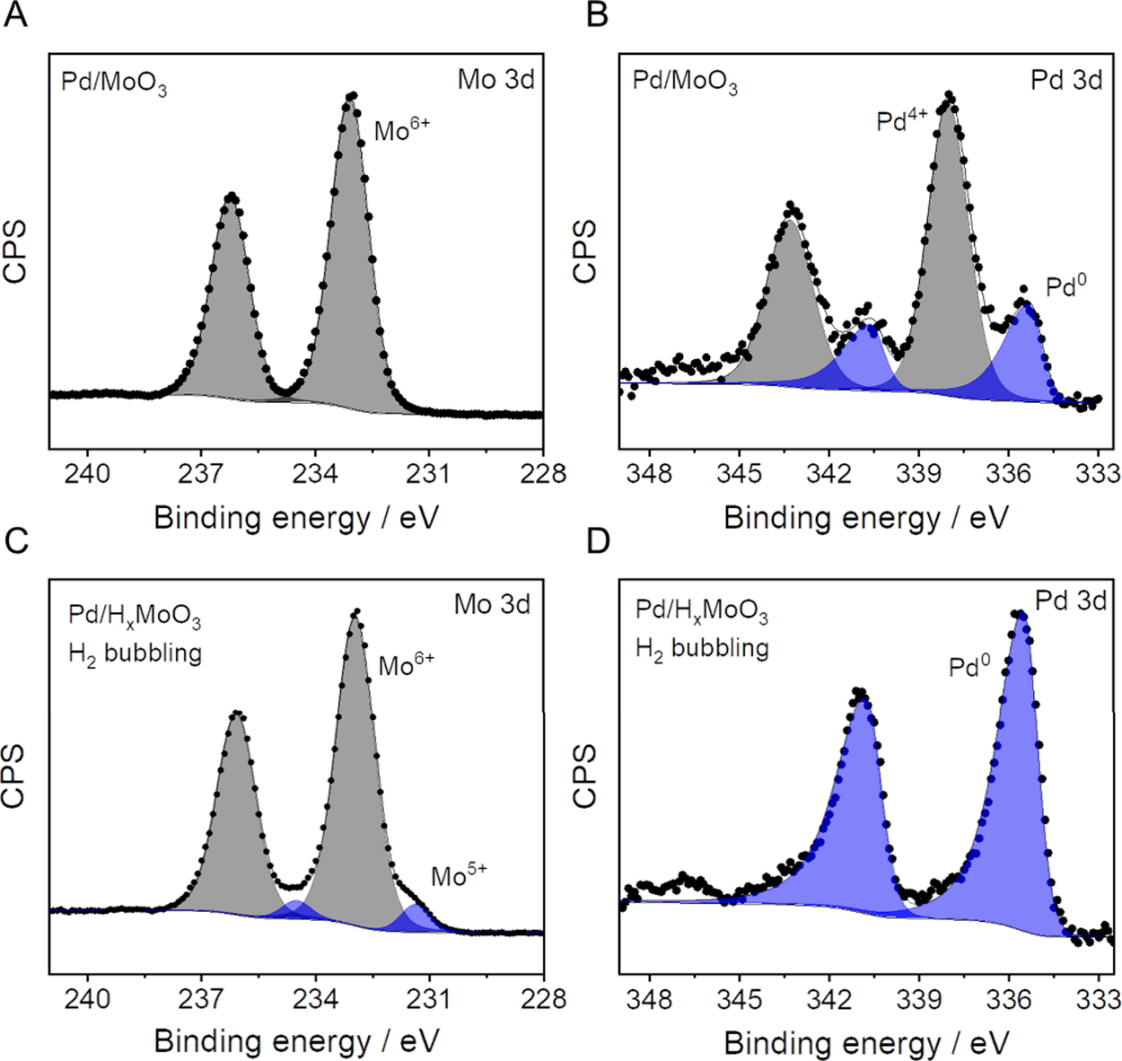
XPS characterization for Pd/MoO_3_ and Pd/H_*x*_MoO_3_. High-resolution photoelectron spectra of (A and B) Pd/MoO_3_ and (C and D) Pd/H_*x*_MoO_3_ in the Mo 3d (A, C) and Pd 3d (B, D) regions.

Figure 3 shows the Mo 3d and Pd 3d core-level X-ray photoelectron spectra of Pd/MoO_3_ (before H_2_ bubbling, Figure 3A,B) and Pd/H_*x*_MoO_3_ (after H_2_ bubbling, Figure 3C,D). The Mo 3d region in the spectra of Pd/MoO_3_ (Figure 3A) can be readily fitted with a pair of symmetric components with peaks at 233.0 and 236.2 eV that correspond to the spin–orbit doublet characteristic of Mo (VI). The Pd 3d region (Figure 3B) is more complex, featuring four relative maxima that can be fitted with two pairs of Pd 3d spin–orbit doublets with peaks at 335.1 and 340.4 eV for one pair and at 337.9 and 343.2 eV for the second pair. These positions are characteristic of metallic Pd and Pd (IV) as in PdO_2_, respectively. Although the low spectral signal makes the quantification less reliable, we estimate from the relative peak areas that 25% of the Pd is in the metallic state. This result indicates that under our employed conditions, the mechanochemical process does not drive the complete reduction or conversion of the Pd precursor to metallic Pd. After H_2_ bubbling (Pd/H_*x*_MoO_3_), the Mo 3d spectrum (Figure 3C) displays a clear shoulder toward low binding energy values. This indicates the presence of a second component that can be fitted with an additional pair of spin–orbit doublets with positions at 231.4 and 234.5 eV. The position of this component is too high to associate it with Mo (IV), and it has been rather associated with the presence of Mo (V).^45^ This partial reduction of Mo (IV) to Mo (V) occurs because of the hydrogen spillover process due to H incorporation in this system. The Pd 3d spectrum for Pd/H_*x*_MoO_3_ (Figure 3D) can be well-fitted with a single pair of asymmetric components (at 335.1 and 340.4 eV) associated with metallic Pd, which indicates the complete reduction of Pd species to the metallic state under the H_2_ bubbling step. The core-level spectra of the O 1s and C 1s are shown in Figure S3. Although no significant changes were detected in the core-level spectra of the O 1s after the H_2_ bubbling step, an increase in the CO_2_ chemisorption capacity of the sample was detected. This behavior has been also observed in MoO_3-x_ samples.^46,47^ The XANES spectrum for Pd/H_*x*_MoO_3_ (Figure S2) shows a similar spectral profile relative to the Pd/MoO_3_ and MoO_3_ samples. This suggests that Mo^5+^ sites are probably concentrated at the surface of the particles, being detected in XPS but not in XANES.

Our spectroscopic data (Figure 1C,D) show that the H_*x*_MoO_3_ material presents both band gap and LSPR excitations, which are in different spectral regions. Whereas the wavelength for band gap excitation corresponds to 433 nm, the LSPR excitation band has its maximum intensities above 600 nm. In addition, Pd NPs present interband transitions in the range of less than 450 nm. Thus, in the next step, we were interested in (*i*) using different wavelengths to investigate and compare how the H_*x*_MoO_3_ LSPR excitation (>600 nm) or a combination of H_*x*_MoO_3_ band gap and Pd interband excitation (<450 nm) influences catalytic activities and (*ii*) using two wavelengths (dual excitation at both <450 and >600 nm) to investigate how the triple play of H_*x*_MoO_3_ band gap, H_*x*_MoO_3_ LSPR, and Pd interband excitation influences catalytic activities. To address this challenge, we started by employing a model catalytic transformation that is catalyzed by Pd only (and thus H_*x*_MoO_3_ has negligible activity) and two different light irradiation wavelengths: 427 nm for the excitation of the H_*x*_MoO_3_ band gap and Pd interband transitions and 640 nm for the excitation of the H_*x*_MoO_3_ LSPR. It is noteworthy that we were interested in using only light in the visible region as the excitation source.

**Figure 4:**
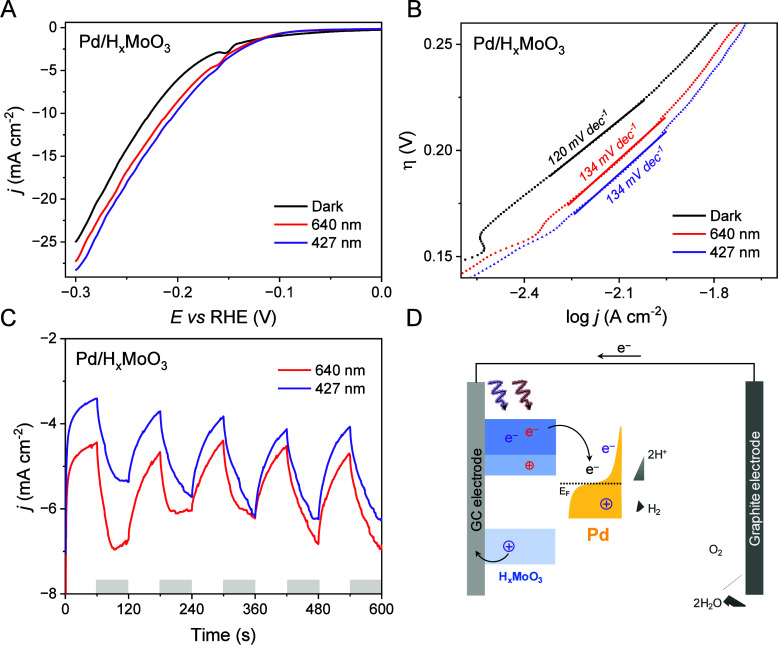
Electrocatalytic performance toward the HER under different light excitation conditions. (A) LSV curves for Pd/H_*x*_MoO_3_ performed in the dark and under 427 and 640 nm LED irradiation conditions recorded at 5 mV s^–1^, (B) Tafel plots calculated from LSV curves, (C) chronoamperometries recorded at −0.2 V under chopped illumination in Ar-saturated H_2_SO_4_ 0.5 M solution, and (D) schematics illustrating the photoelectrochemical HER using Pd/H_*x*_MoO_3_ as photocathode and graphite electrode as counter electrode.

Figure 4A shows the LSVs for Pd/H_*x*_MoO_3_ using dark (black traces) and light irradiation conditions at 427 or 640 nm (violet and red traces, respectively). The LSV curves indicate that the catalytic activity increases to a similar extent under both light illumination conditions. The overpotential at a current density of 10 mA cm^–2^ (η_10_), which is a benchmark of HER performance, was measured. The lowest η_10_ values (and thus higher activities) were obtained under light irradiation, corresponding to 202 and 210 mV under 427 and 640 nm irradiation, respectively. Under dark conditions, the overpotential was 227 mV. The decrease in overpotential as well as the increase in current density for Pd/H_*x*_MoO_3_ at both wavelengths indicates that both H_*x*_MoO_3_ LSPR excitation and H_*x*_MoO_3_ band gap excitation and Pd interband transitions can contribute to enhancing HER activity, and these two effects led to a similar enhancement. The LSVs for pure H_*x*_MoO_3_ shown in Figure S4A also revealed an increase in current density under light irradiation for both wavelengths, with a 2-fold enhancement compared to dark conditions. This agrees with the band gap and LSPR excitation in the H_*x*_MoO_3_. However, high overpotential values for pure H_*x*_MoO_3_ were detected as expected due to its low catalytic activity for the HER.^48^

The Tafel plots presented in Figure 4B can provide important information about the HER reaction mechanism and rate-determining step. The Tafel slopes of 120, 134, and 134 mV dec^–1^ for Pd/H_*x*_MoO_3_ in the dark and at 640 and 427 nm, respectively, were in good agreement with the reported values of Pd-modified electrodes.^49−51^ The values suggest that the Volmer step (H^+^ + e^–^ + Pd ⇌ Pd–H_ad_) is the rate-determining step.^52^ Chronoamperometric experiments under chopped light conditions for Pd/H_*x*_MoO_3_ and the resultant photocurrent transients are reported in Figure 4C. The photocurrent generated under 427 and 640 nm irradiation promoted an average increase of 2.1 and 1.9 mA in current densities, respectively. A fast and reproducible current response to on–off illumination cycles was detected for both wavelengths, in agreement with the participation of photogenerated electrons from band gap excitation, interband, and LSPR excitation over the current enhancements. A similar effect was observed for H_*x*_MoO_3_ as shown in Figure S4B.

These results show that both the H_*x*_MoO_3_ LSPR excitation and the H_*x*_MoO_3_ band gap excitation coupled with Pd interband transitions play important roles in enhancing the Pd catalytic activity toward the HER as illustrated in Figure 4D. Band gap excitation coupled with interband transitions (violet dashed line) leads to the formation of excited electrons that can be transferred to the Pd sites that, together with hot electrons from the interband transition, facilitate the HER process by reducing protons (H^+^) to hydrogen (H_2_). LSPR excitation in H_*x*_MoO_3_ at 640 nm leads to the formation of hot electrons and holes. Here, hot electrons can be transferred to the Pd sites, also facilitating the HER by accelerating the reduction of H^+^ species. Thus, the similar HER performances under both 427 and 640 nm illumination indicate a similar effect of the LSPR excitation in H_*x*_MoO_3_ as compared to the H_*x*_MoO_3_ band gap excitation coupled with Pd interband transitions. It is important to note that thermal effects (localized heating) under light illumination due to plasmonic excitation may also contribute to the enhanced HER activities, and the elucidation of thermal vs nonthermal effects in plasmonic catalysis is important.^53,54^ Nevertheless, the effects of excited or hot charge carriers relative to photothermal heating are difficult to disentangle under our experimental conditions, and it is plausible that both of these effects are contributing to the enhanced catalytic activities. Therefore, visible light excitation at 427 and 640 nm offers complementary mechanisms for improving the catalytic activity in the HER to a similar extent in Pd/H_*x*_MoO_3_. The similar enhancements observed at both wavelengths may indicate that better charge generation and utilization occur under plasmonic excitation (640 nm) under our conditions relative to Pd and H_*x*_MoO_3_ interband electrons. However, this is difficult to evaluate precisely, as 427 nm does not correspond to the maximum absorption region for H_*x*_MoO_3_. It is noteworthy that the HER performances can be further improved in the future by optimizing the Pd loading onto the electrode and optimizing the synthesis.

DFT calculations on the electronic properties of Pd/H_*x*_MoO_3_ model NPs and the difference in Gibbs free energies (Δ*G*_H*_) for the H* adsorption/desorption relative to Pd and H_*x*_MoO_3_ model NPs were performed to obtain further insights into the detected HER activity (Figures S5–S7, see Supporting Information for further details).^55,56^ The simulation data agree with the boosting of HER activity in Pd/H_*x*_MoO_3_ via the optimization of adsorption/desorption of H* species (under both dark and light conditions) and the facilitation of LSPR-excited charge carrier transfer (from H_*x*_MoO_3_) to Pd NPs under light excitation.^56^

**Figure 5:**
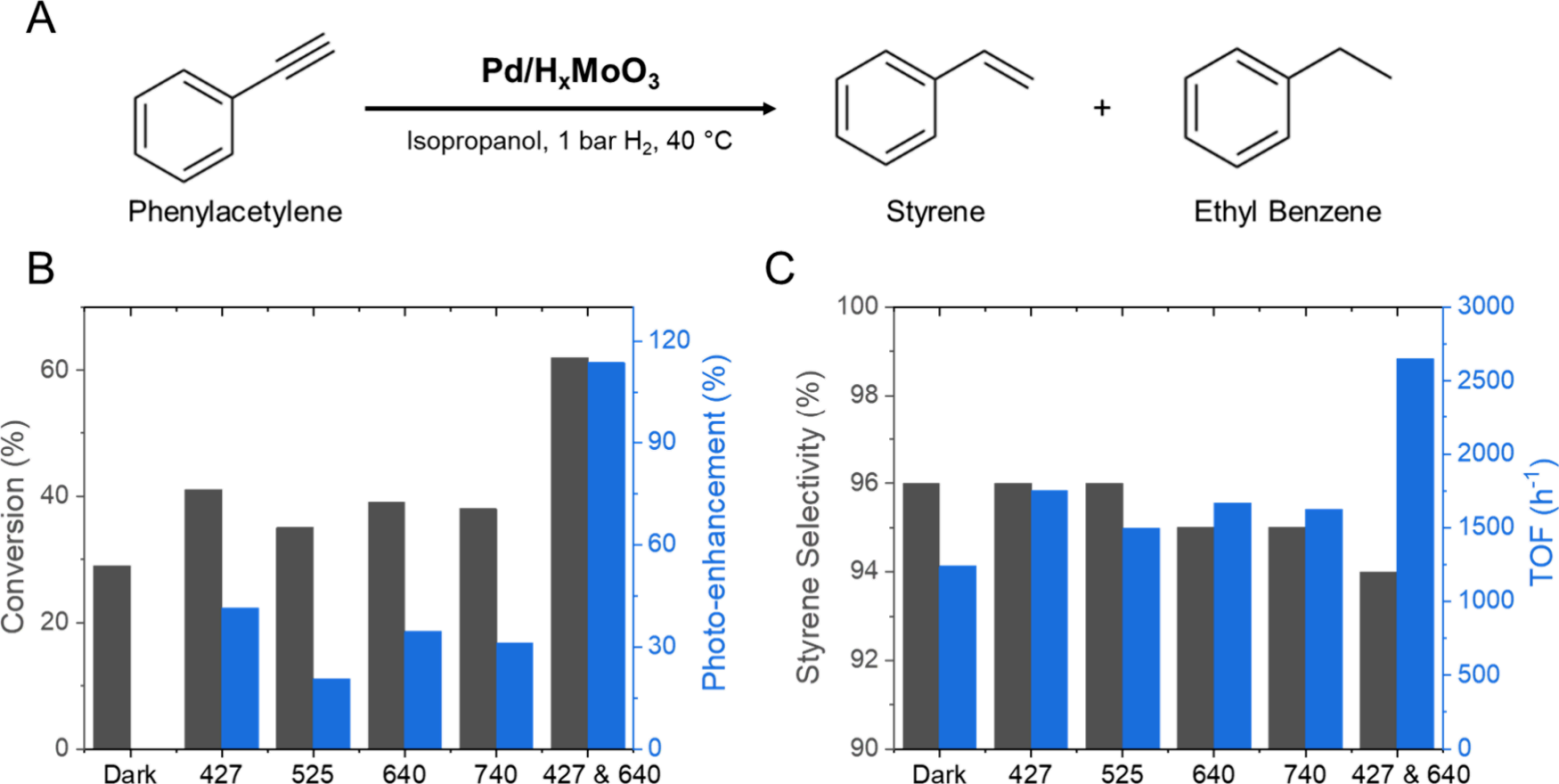
Photocatalytic hydrogenation of phenylacetylene over Pd/H_*x*_MoO_3_ catalyst in the dark and under 427, 525, 640, and 740 nm LED irradiation conditions. (A) Reaction scheme, (B) total conversion and photoenhancement, and (C) selectivity for the hydrogenation of phenylacetylene to styrene and turnover frequency (TOF). The reactions were conducted under an H_2_ atmosphere (1 bar) with 10 mL of isopropanol, 0.5 mmol of the substrate, and 1.5 mg of catalyst. The reaction time was 1 h.

We then employed the hydrogenation of phenylacetylene as a model transformation, as depicted in Figure 5A, to investigate the effects of different light wavelengths as well as dual excitation at 427 and 640 nm over catalytic activity and reaction selectivity (our HER setup does not enable the use of dual excitation conditions). In this transformation, two products are possible: styrene (from semihydrogenation) and ethylbenzene (from full hydrogenation). Moreover, it is well established that Pd NPs have good activity toward alkyne hydrogenation reactions, whereas H_*x*_MoO_3_ is not active. Therefore, as in the HER, the detected activities can be assigned to Pd.^57,58^

Figure 5B (and Table S1) shows the phenylacetylene conversion % and the calculated photoenhancement percentage for the Pd/H_*x*_MoO_3_ catalyst under different light irradiation wavelengths. GC and GC–MS analyses of the products are shown in Figures S8 and S9. The conversion % under dark conditions corresponded to 29%. Under light excitation conditions, an enhancement in the conversion % was observed for all the wavelengths and corresponded to 41, 39, 38, and 35% under 427, 640, 740, and 525 nm, respectively. This suggests relatively similar conversion percentages under light excitation as a function of the wavelengths, with the highest and lowest values corresponding to 427 and 525 nm, respectively. The highest photoenhancement was observed for 427 nm excitation, corresponding to 41%, which can be assigned to the band gap excitation of H_*x*_MoO_3_ and interband transitions on Pd. Here, as discussed for the HER, visible excitation can lead to energetic electrons, which can contribute to accelerating the phenylacetylene hydrogenation reaction.^59,60^ According to our previous data on antenna–reactor plasmonic catalysts with Pd, the first hydrogenation step corresponds to the rate-determining step, and the excited electrons or hot electrons can contribute to accelerating this process.^59^

Regarding the selectivity, all dark and light-irradiation conditions led to ≥95% selectivity for the formation of styrene (semihydrogenation reaction), as illustrated in Figure 5C, indicating that our catalyst was selective. The observed selectivity to styrene can be attributed to a two-step hydrogenation process. Initially, the phenylacetylene undergoes partial hydrogenation, leading to the formation of styrene. Subsequently, styrene undergoes further hydrogenation to produce ethylbenzene. As we maintained the conversion levels up to 60%, the reaction predominantly yielded styrene as the major product. This is supported by our experiments, where we expanded the scope of substrates for hydrogenation (Table S2). In instances where the substrate underwent a higher catalytic conversion (Table S2, entry 4), the fully hydrogenated product was detected. We calculated the turnover frequency (TOF) based on the Pd loading (0.83 wt %). The results are also presented in Figure 5C. TOF values ranged from 1239 in the dark to 1752 h^–1^ under 427 nm excitation. A comparison of the estimated TOF and other reported Pd-based catalysts is presented in Table S3.

After separately investigating the effect of distinct wavelengths over enhanced catalytic activities and selectivity under light excitation, which enabled us to separate the effect of H_*x*_MoO_3_ LSPR from the H_*x*_MoO_3_ band gap and Pd interband transition, we decided to unravel whether we could employ these three effects at the same time to achieve superior performance under dual excitation condition. In other words, we were interested in investigating the combined effect of the band gap and LSPR excitation from H_*x*_MoO_3_ as well as the interband transitions from Pd over the catalytic activities and selectivity. This was achieved by employing both 427 and 640 nm as the light irradiation wavelengths to excite all of these processes.

**Figure 6:**
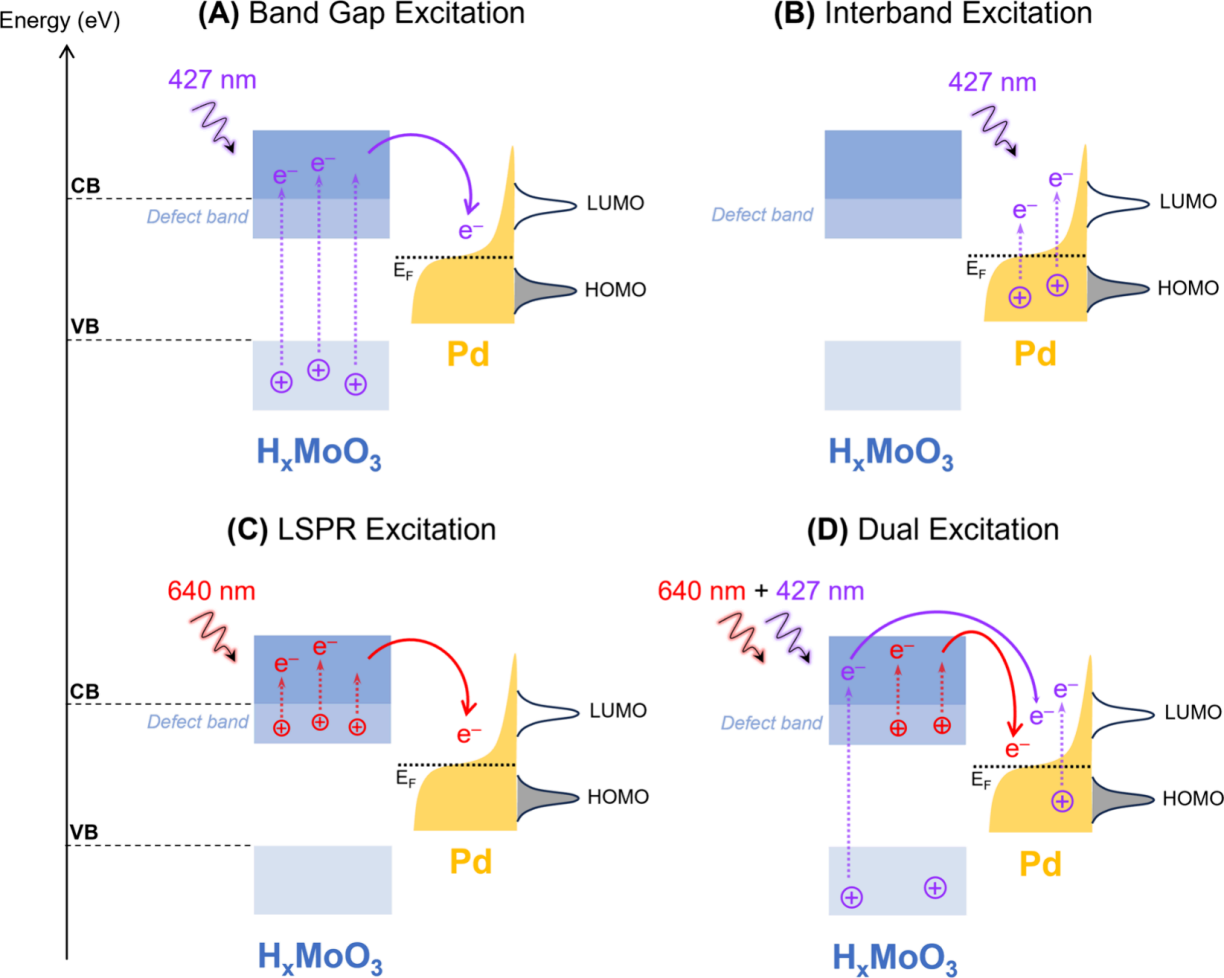
Proposed scheme for the energy diagram and optical excitations for Pd/H_*x*_MoO_3_. (A) H_*x*_MoO_3_ band gap excitation at 427 nm; (B) Pd interband transitions at 427 nm; (C) H_*x*_MoO_3_ LSPR excitation (640 nm); and (D) triple play of the H_*x*_MoO_3_ band gap and LSPR excitation and Pd interband transitions under dual illumination.

Interestingly, by employing the dual excitation at 427 and 640 nm, a significant increase in the conversion % from 41% (under the best light irradiation conditions achieved at 427 nm) to 62% was detected. This corresponds to a 114% photoenhancement and a TOF of 2650 h^–1^, with a selectivity toward styrene of 94%. This result shows that the dual excitation and thus the triple play of the H_*x*_MoO_3_ band gap and LSPR excitation and Pd interband transitions lead to an enhancement in conversion %, which is superior to the sum of excitation at 427 or 640 nm only. This concept could be applied to a variety of substrates as shown in Table S2, suggesting the versatility of this approach. Figure 6 summarizes the roles of the H_*x*_MoO_3_ band gap and LSPR excitation and Pd interband transitions over the enhanced catalytic activities under 427, 640, and dual 427 + 640 nm excitation conditions. When 427 nm excitation is employed (Figure 6A,B), light absorption occurs via band gap excitation on H_*x*_MoO_3_ (Figure 6A) and interband transitions in Pd (Figure 6B). This leads to the formation of energetic electrons from Pd and from H_*x*_MoO_3_, which can be transferred to Pd. Both of these effects contribute to accelerating the phenylacetylene hydrogenation reaction by activating adsorbed molecules (phenylacetylene or H-species) via electronic or vibrational excitation, accelerating the first hydrogenation step (rate-determining step). When 640 nm excitation is employed, as shown in Figure 6C, LSPR excitation in H_*x*_MoO_3_ generates hot electrons that are then transferred to the Pd NPs, also leading to enhanced activities. Finally, when both 427 and 640 nm are employed as light irradiation wavelengths (Figure 6D), all these processes, i.e., band gap and LSPR excitation from H_*x*_MoO_3_ and interband transitions from Pd, lead to the formation and transfer of energetic electrons to the Pd sites, leading to a superior enhancement in catalytic activity relative to the separate effect of each of these light wavelengths. These results demonstrate that the control and optimization over the light irradiation and optical excitation processes using dual excitation conditions can lead to the maximization of catalytic activities in the visible range, opening new avenues for the optimization of photocatalytic and plasmonic properties.

Finally, we performed DFT calculations to gain insights into the observed activity toward the hydrogenation of phenylacetylene (Figures S10–S15). Figure S10 shows that phenylacetylene adsorbs at the Pd and Pd/H_*x*_MoO_3_ surfaces in a flat configuration relative to Pd due to the interaction of the aromatic ring with the surface.^59^ The PDOS for phenylacetylene before and after adsorption is shown in Figure S11A and reveals the strong interaction at the surface with the downshifted 2p orbitals. The calculated charge density differences (Figure S11B) agree with this observation and suggest a stronger local charge redistribution relative to the pure Pd (111) model (Figure S12). To quantitatively estimate the interaction between Pd sites and adsorbed phenylacetylene, the projected crystal orbital Hamilton population (pCOHP) of the Pd–C bond (Pd in the surface site and terminal C in adsorbed phenylacetylene, Figure S13) was examined. The integral COHP value (ICOHP) could be calculated by integrating the partial COHP below the *E*_F_, suggesting the number of bonded electrons between the selected Pd and C atoms and the corresponding bonding strength.^56^ It was clear that the ICOHP of Pd–C in Pd/MoO_3-x_ was larger than that of Pd (111) and indicated stronger phenylacetylene adsorption (Figure S11C).

The calculated hydrogenation reaction pathway and changes in energy for the formation of styrene and ethylbenzene are shown in Figures S11D and S14. The calculated maximum energy barriers for the hydrogenation of phenylacetylene to styrene on Pd/H_*x*_MoO_3_ and Pd (111) models were 0.46 and 0.68 eV, respectively. To achieve selective hydrogenation toward styrene, the desorption of adsorbed styrene (PhCHCH_2_*) is key. As shown in Figure S11D, the styrene desorption energy on Pd/H_*x*_MoO_3_ is further reduced relative to that on Pd (111) (0.70 and 1.14 eV, respectively), indicating that the presence of the H_*x*_MoO_3_ further contributes to driving the selectivity for semihydrogenation in this system. The simulations of the charge density difference results (Figure S15) also indicate that the adsorption of styrene over Pd (111) is stronger than that on Pd/H_*x*_MoO_3_. Finally, as described for the HER, it is plausible that thermal effects may also contribute to the enhanced reaction rates under light excitation conditions.

## Conclusions

We report herein the investigation and optimization of photocatalytic properties in the visible region by controlling different optical excitation processes via distinct light illumination conditions. To this end, we employed Pd NPs supported onto H_*x*_MoO_3_ (Pd/H_*x*_MoO_3_) as a model catalyst, as this system supports band gap and LSPR excitation (from H_*x*_MoO_3_) as well as interband transitions (from Pd). The Pd/H_*x*_MoO_3_ was obtained by hydrogen spillover of Pd/MoO_3_, which was prepared by a one-pot mechanochemical synthesis under solventless conditions. Whereas the H_*x*_MoO_3_ band gap excitation and Pd interband transitions can be excited at 427 nm, the H_*x*_MoO_3_ LSPR excitation is maximized at >600 nm. Therefore, by employing 427 nm as the excitation wavelength, we could probe the effect of H_*x*_MoO_3_ band gap excitation and Pd interband transitions over catalytic activities, and by employing 640 nm, we could probe the effect of the LSPR excitation. We employed the HER and the phenylacetylene hydrogenation as model transformations to study how these different excitation conditions affect the catalytic activities from Pd (catalytic sites). Our data show that either 427 or 640 nm excitation led to similar enhancements in catalytic activity for both transformations and that the hydrogenation of phenylacetylene was selective for the formation of styrene. Interestingly, we have found that the catalytic activity toward the phenylacetylene hydrogenation was maximized when we employed dual excitation conditions, i.e., employing both 427 and 640 nm as the light irradiation conditions. In this case, an increase in the photoenhancement to 110% was achieved relative to 41% (under 427 nm irradiation) and 39% (under 640 nm irradiation). Moreover, the catalyst was still selective toward the formation of styrene (94%). This increase in catalytic activity under dual light excitation conditions was assigned to the triple play of the H_*x*_MoO_3_ band gap, H_*x*_MoO_3_ LSPR, and Pd interband excitation. These processes optimize and maximize the formation of energetic electrons at the catalytic sites (Pd) under light illumination conditions, allowing for the superior acceleration of the phenylacetylene hydrogenation. We believe that the results reported herein provide important insights for the future design and development of higher performing photocatalysts, in which the ability to control optical excitations can provide an important avenue to maximize catalytic activities. Coupled with green and solventless catalyst synthesis methods that enable large-scale production, these findings can bring solar-driven chemistry for a sustainable planet one step closer to reality.

## Methods

### Reagents and Instrumentation

Molybdenum(VI) oxide powder (MoO_3_, 99.5%, Sigma-Aldrich, >1 μm in particle size), potassium palladium(II) chloride (K_2_PdCl_4_, 99.9%, Sigma-Aldrich), isopropanol (C_3_H_8_O, HPLC grade 99.9%, Sigma-Aldrich), acetone (CH3COCH3, 99%, Honeywell), sulfuric acid (H_2_SO_4_, ACS reagent 98%, Honeywell), Nafion perfluorinated resin (10 wt % in H_2_O, Sigma-Aldrich), absolute ethanol (CH_3_CH_2_OH, 99.9%, Honeywell), sodium borohydride (NaBH_4_, 98%, Fisher Chemical), and carbon black Vulcan XC-72R (Fuel Cell Store) were used. All chemicals were used without further purification. Deionized water (18.2 MΩcm, Milli-Q) was used throughout the experiments.

Transmission electron microscopy (TEM) measurements were conducted on a JEOL JEM-2200FS microscope operating with an accelerating voltage of 200 kV. The samples were prepared by drop casting an aqueous suspension onto a carbon film on a copper TEM grid (400 square mesh) and drying under ambient conditions. Powder X-ray diffraction (PXRD) analysis was performed on a Bruker D8 Advance diffractometer with a Cu Kα radiation source (λ = 1.5406 Å) and a Ni filter. The diffraction patterns were collected in the 2θ range of 10–70°, with a 0.02° step width and 1 s/step count time. The calculation of the crystallite size was performed by the Scherrer equation:
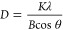
where *D* is the average crystallite size, *K* is the shape factor (adopted to 0.94 for roundish particles), *B* is the fwhm in radians of a peak at the angle theta, and lambda is the wavelength (0.154 nm for Cu Kα).

Atomic emission spectroscopy (AES) analysis was performed on an Agilent Technologies 4100 MP spectrometer to determine the Pd content. Pd standard solutions of 1, 2, 5, 7, and 10 ppm were prepared using K_2_PdCl_4_ salt dissolved in 0.5 M HCl solution. The catalyst was dissolved in *aqua regia* followed by the evaporation of the solvent on a hot plate at 50 °C. Then the catalyst was redispersed in 0.5 M HCl solution, resulting in a Pd concentration of 5 ppm. UV–vis analysis was conducted in a Shimadzu UV-2600 PC spectrophotometer using quartz cuvettes with an optical path of 1 cm. The sample was dispersed in isopropanol and purged for 5 min with H_2_ before the measurement. The photoluminescence (PL) spectra of the catalysts (powder samples) were recorded at room temperature using a Horiba FluoroMax-4 Spectrofluorometer equipped with a 150 W xenon arc lamp as the excitation source and a Front Face sample holder. All the spectra were recorded at room temperature.

X-ray photoelectron spectra were recorded with a lab-based spectrometer (SPECS GmbH, Berlin) using a monochromated Al Kα source (*h*ν = 1486.6 eV) operated at 50W as the excitation source. In the spectrometer, the X-ray was focused with a μ-FOCUS 600 monochromator onto a 300 μm spot on the sample, and the data were recorded with a PHOIBOS 150 NAP 1D-DLD analyzer in fixed analyzer transmission (FAT) mode. The pass energy was set to 40 eV for the survey scans and 20 eV for the high-resolution regions. The binding energy scale was calibrated using Au 4f_7/2_ (84.01 eV) and Ag 3d_5/2_ (368.20 eV). Charge compensation was required for data collection. Recorded spectra were additionally calibrated against the C 1s internal reference. Data interpretation was performed with Casa XPS. Shirley or two-point linear background was used depending on the spectrum shape.

The X-ray absorption near edge spectra (XANES) were measured using the Rowland circle spectrometer Hel-XAS^61^ of the Helsinki Center for X-ray Spectroscopy equipped with a spherically bent Si (12,12,0) crystal analyzer (bending radius 0.5 m). The Bremsstrahlung from a water-cooled Ag-anode X-ray tube was monochromated and focused by the crystal onto a Si drift diode detector. Scanning the spectrometer Bragg angle across the Mo K absorption edge energy yielded the intensity *I*(*E*) with and *I*_0(*E*) without the sample, from which the dimensionless XANES spectrum \mu(*E*) × *d* was obtained, *d* being the sample thickness. A linear pre-edge background was subtracted from the spectra, and the spectra were normalized to equal area.

### Synthesis of Pd/MoO_3_ and Pd/H_*x*_MoO_3_

The synthesis of supported Pd nanoparticles in MoO_3_ was prepared by partial reduction of the Pd precursor and commercial MoO_3_ using sodium borohydride (NaBH_4_) in a ball-milling device following a previous method.^21^ The mechanochemical synthesis was performed on a vertical vibratory ball mill (Pulverisette 23, Fritsch) at 50 Hz using a PMMA milling jar (10 mL) with a single zirconia milling ball (diameter of 10 mm; 3.14 g). In a typical procedure, commercial MoO_3_ (974 mg) and K_2_PdCl_4_ (10 mg) were milled for 10 min to achieve a good dispersion; then NaBH_4_ (26 mg) was added to the jar, and the mixture was milled for 1 h. The resultant Pd/MoO_3_ powder was washed with water and ethanol by centrifugation, resuspension, and removal of the supernatant several times. The final product was dried at 80 °C for 12 h. For comparison, commercial MoO_3_ (974 mg) and NaBH_4_ (26 mg) were ball milled for 1 h without the Pd precursor, and the resultant MoO_3_ powder was washed and dried following the same procedure described above. Pd/H_*x*_MoO_3_ NPs were prepared by bubbling a suspension of the obtained Pd/MoO_3_ (5g/L) with H_2_ gas for 5 min at 1 atm.

### Electrocatalytic Tests: Hydrogen Evolution Reaction (HER)

A water-jacketed three-electrode glass cell was used during the electrochemical experiments, and the temperature was kept at 25 °C by using a water bath and a refrigerated circulator (Julabo F12-MA). A glassy carbon electrode (6 mm diameter, geometric area of 0.2827 cm^2^) was used as a working electrode, a graphite rod was used as a counter electrode, and a reversible hydrogen electrode (RHE) was used as a reference electrode. The GC electrode was cleaned by polishing with alumina slurry (0.05 μm) and an ultrasonic bath in ultrapure water and acetone for 5 min each. Then 5 mg of the catalyst and 1 mg of carbon Vulcan XC-72R were dispersed in 1 mL of isopropanol/water (3:1) solution (containing 0.1 wt % Nafion) to form a homogeneous ink, and 30 μL of the dispersion was dropcast on the GC electrode. The total catalyst loading was 500 μg/cm^2^, and the Pd loading for the Pd/MoO_3_ catalyst was ≈4 μg/cm^2^. Linear sweep voltammetry (LSV) measurements were performed at a scan rate of 5 mV s^–1^, and chronoamperometry (CA) measurements were recorded at −0.2 and −0.5 V for Pd/MoO_3_ and MoO_3_, respectively, both in Ar-saturated 0.5 M H_2_SO_4_ solution. For photoelectrocatalytic experiments, the cell was irradiated by Kessil PR 160L LED with 640 or 427 nm irradiation, resulting in a total irradiance of 120 or 142 mW cm^–2^, respectively. The electrochemical measurements were carried out using an Autolab PGSTAT 128N equipped with a Scan 250 modulus potentiostat. Before the experiments, the solution was purged with Argon 2.2, and during the data collection, this gas was kept in the cell headspace.

### Catalytic Tests: Hydrogenation of Phenylacetylene

The hydrogenation of phenylacetylene was performed using a 100 mL Schlenk flask. Initially, 1.5 mg of the Pd/MoO_3_ catalyst and 0.5 mmol of phenylacetylene were suspended in isopropanol, reaching a total volume of 10 mL, and the flask was kept for 10 min in an ultrasound bath to obtain a homogeneous dispersion. The mixture was purged with hydrogen for 5 min, and a balloon filled with H_2_ at a pressure of 1 bar was used to seal the flask. Two flasks were prepared at the same time to run the reactions under dark and light conditions separately. For the dark (light off) experiment, the reactor was covered with aluminum foil. For the irradiation experiment (light on), four Kessil PR 160L LEDs with 427, 525, 640, or 740 nm irradiance (Kessil PR 160) were positioned 6 cm away from the reactor, resulting in a total irradiance of 14.34 mW/cm^2^ according to the experimental setup reported elsewhere.^62^ A cooling fan was also placed above the reactor to avoid overheating. The experiments with two different wavelengths were performed using two 427 and two 740 nm LEDs in a similar configuration and total irradiance as described above. For all experiments, the reactors were kept at 40 °C in a silicon bath, the solutions were stirred using a Teflon-coated magnetic stirrer, and the reaction was carried out for 1 h.

Gas chromatography (GC) was performed at the end of the reaction to analyze the products. Two aliquots of 1.5 mL were collected from each reactor, centrifugated for 10 min at 13,000 rpm to remove the catalyst, and filtered using syringe filters (0.22 μm filter diameter). Afterward, the liquid sample was injected in a NEXIS GC-2030 gas chromatograph coupled to a flame ionization detector and a Crossbond SH-Rxi-5 ms capillary column (30 m × 0.25 mm × 0.25 μm), allowing for the separation and quantification of the reaction products. The heating program used during the analysis was as follows: 70 °C for 1 min, 6 °C min^–1^ until 85 °C, and then 100 °C min^–1^ until 300 °C. For the *t* = 0 samples, a solution containing 0.5 mmol of phenylacetylene in isopropanol was injected before each catalytic test using the same procedure described above.

The phenylacetylene conversion (*X*_PA_%) was calculated using eq 1:
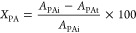
1where *A*_PAi_ and *A*_PAt_ are the chromatographic areas of phenylacetylene in the samples at time = 0 and *t*, respectively. The product selectivity was calculated using eq 2:
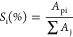
2where *A*_pi_ and ∑*A*_j_ are the chromatographic area of the product p_i_ and the sum of the chromatographic area of all products, respectively. The turnover frequency (TOF) was estimated by dividing the number of moles of reactant converted per number of active sites per time.

### Computational Methods

DFT simulations were run with the first-principles simulation DMol3 module of Materials Studio.^63^ The generalized gradient approximation of Perdew–Burke–Ernzerhof exchange-correlation functionals was adopted for all the calculations.^64^ Core treatment was adopted as all electron to conduct the metal relativistic effect, and the double numerical plus polarization function basis set was used. A smearing of 0.01 Ha to the orbital occupation and a 1 × 10^–5^ Ha convergence criterion for self-consistent-field (SCF) calculations were applied. The geometry optimization convergence tolerance for energy change, max force, and max displacement were 1 × 10^–5^ Ha, 0.004 Ha/Å, and 0.005 Å, respectively. The vacuum spacing in the direction along the *Z* axis with respect to the surface was 20 Å between neighboring slab images, which is sufficient to eliminate the interactions between the slabs. Activation barriers were obtained based on the linear synchronous and quadratic synchronous transit LST/QST.^65^

The free energy (Δ*G*) calculations of each elementary step were based on the standard hydrogen electrode model,^66^ and the reaction free energy change can be obtained with the equation below:
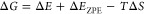
3where Δ*E* is the total energy difference before and after intermediate adsorption and Δ*E*_ZPE_ and Δ*S* are, respectively, the differences in zero-point energy and entropy, respectively.

For the HER, the hydrogen-adsorption Gibbs free energy (Δ*G*_H*_) was calculated according to the equation below:
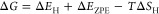
4where Δ*E*_H_ is the hydrogen absorption energy, Δ*E*_ZPE_ is the correction of the zero-point energy, *T* is the temperature setting at 298.15 K, and Δ*S*_H_ is the entropy difference between the absorbed hydrogen atom and free H_2_ molecule, and Δ*E*_ZPE_-*T*Δ*S*_H_ is approximately 0.24 eV.

## Supporting Information Available

Additional characterization and performance evaluation (XRD, XANES, XPS, LSVs), catalytic data (LSVs, substrate scope, and GC and GC–MS analyses), and computational modeling (PDF)
