# Supplementary material for: Triple Play of Band Gap, Interband, and Plasmonic Excitations for Enhanced Catalytic Activity in Pd/HxMoO3 Nanoparticles in the Visible Region
Source: ACS Appl Mater Interfaces. 2024 Feb 21;16(9):11467–78. doi: 10.1021/acsami.3c17101 (PMC11393804; doi:10.1021/acsami.3c17101)
Supplement: Supplementary file 1 — am3c17101_si_001.pdf [file am3c17101_si_001.pdf]

Support Information for

Triple play of band gap, interband, and plasmonic excitations for enhanced catalytic activity in Pd/H_xMoO_3 nanoparticles in the visible region

Leticia S. Bezerra,[1] Samir A. Belhout,[1] Shiqi Wang,[1] Jhon Quiroz,[1] Paulo F.M. de Oliveira,[2] Shwetha Shetty,[1] Guilherme Rocha,[1] Hugo L. S. Santos,[1] Sana Frindy,[1] Freddy E. Oropeza,[3] Víctor A. de la Peña O'Shea,[3] Antti-Jussi Kallio,[4] Simo Huotari,[4] Wenyi Huo,[5,6*] and Pedro H.C. Camargo[1,*]

[1] *Department of Chemistry, University of Helsinki, A.I. Virtasen aukio 1, PO Box 55, FIN-0014 Helsinki, Finland.*

[2] *Departamento de Química Fundamental, Instituto de Química, Universidade de São Paulo. Av. Lineu Prestes 748, 05508000, São Paulo, Brazil.*

[3] *Photoactivated Processes Unit, IMDEA Energy Institute, Avda. Ramón de la Sagra 3, 28935 Mostoles, Madrid, Spain.*

[4] *Department of Physics, University of Helsinki, P.O. Box 64, FI-00014 Helsinki, Finland.*

[5] *College of Mechanical and Electrical Engineering, Nanjing Forestry University. Nanjing, 210037, P. R. China.*

[6] *NOMATEN Centre of Excellence, National Centre for Nuclear Research. Otwock, 05-400, Poland*

* *Corresponding author.* Email: pedro.camargo@helsinki.fi, wyhuo@njfu.edu.cn

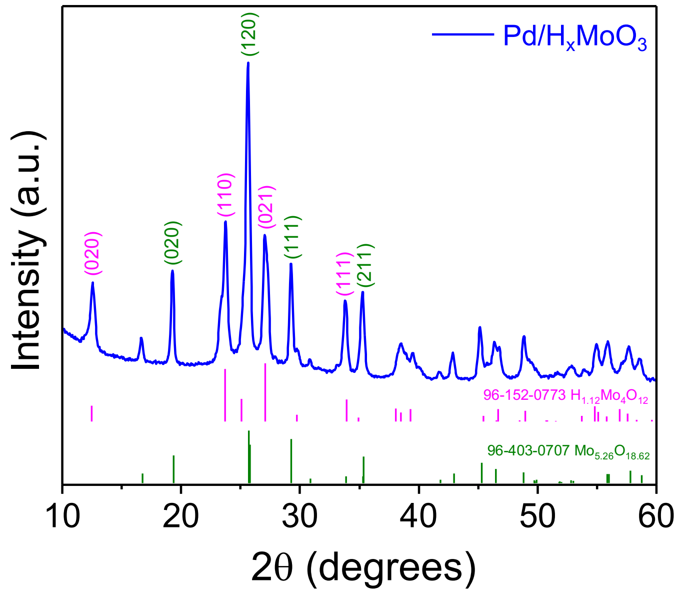

Figure S1. Powder XRD patterns for the Pd/H_xMoO_3 sample.

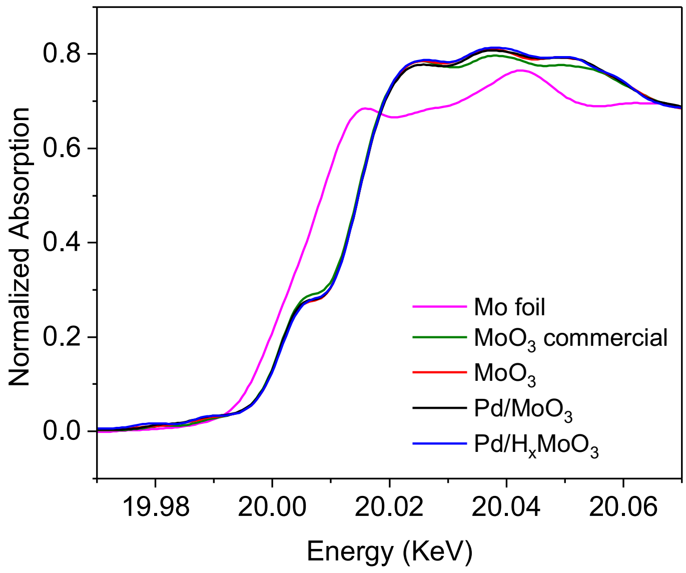

Figure S2. Mo–K edge XANES spectra of MoO_3, Pd/MoO_3, Pd/H_xMoO_3, Mo foil, and commercial MoO_3.

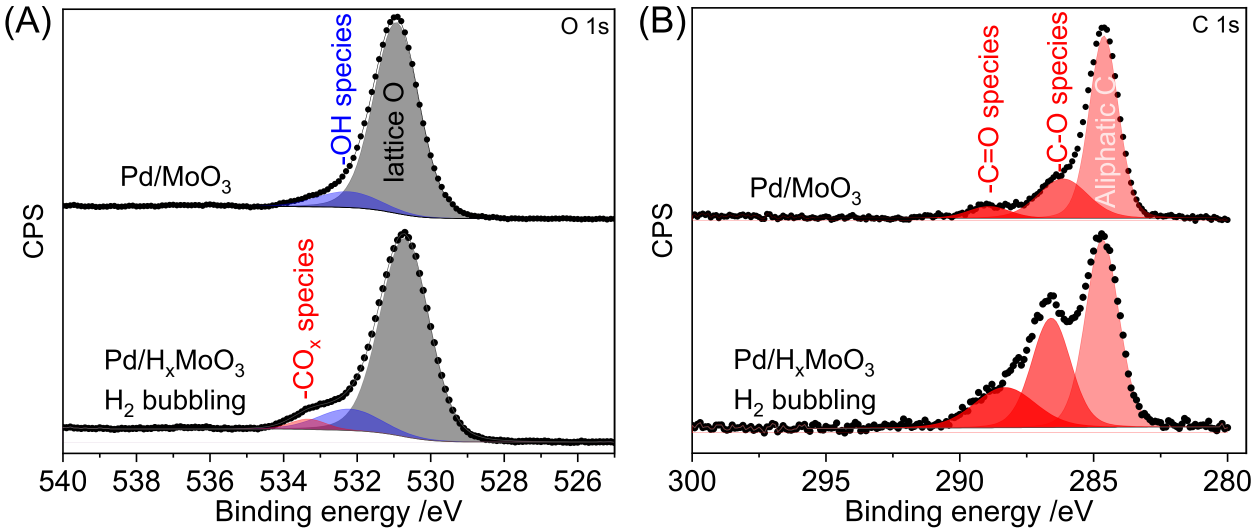

Figure S3. XPS characterization for Pd/MoO_3 and Pd/H_xMoO_3. High-resolution photoelectron spectra in the O 1s (A) and C 1s (B) regions.

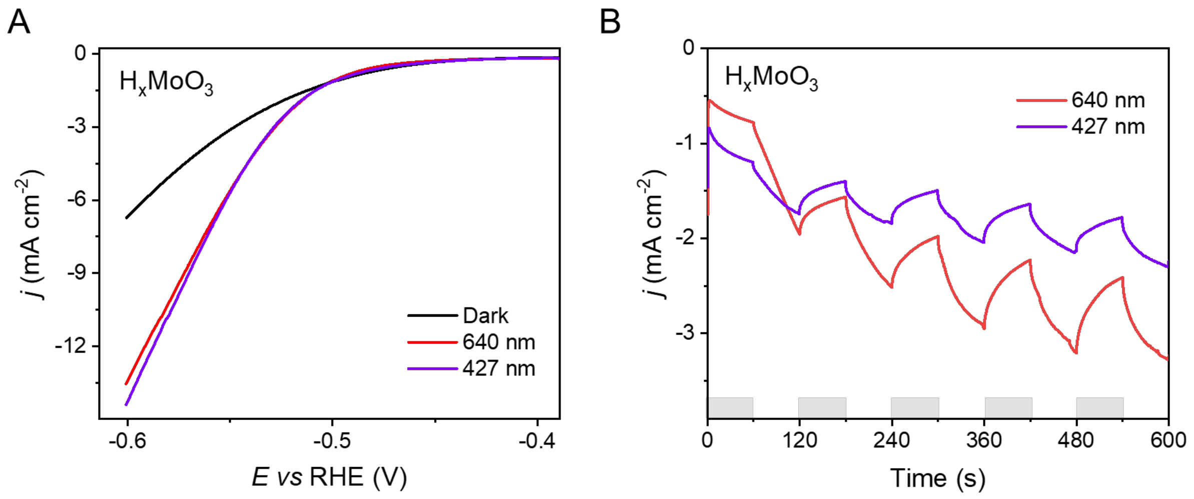

Figure S4. (A) LSV curves for H_xMoO_3 performed in dark and under 427 and 640 nm light irradiation conditions recorded at 5 mVs^{-1}. (B) Chronoamperometry for H_xMoO_3 recorded at -0.5 V under chopped illumination in Ar-saturated H_2SO_4 0.5 M solution.

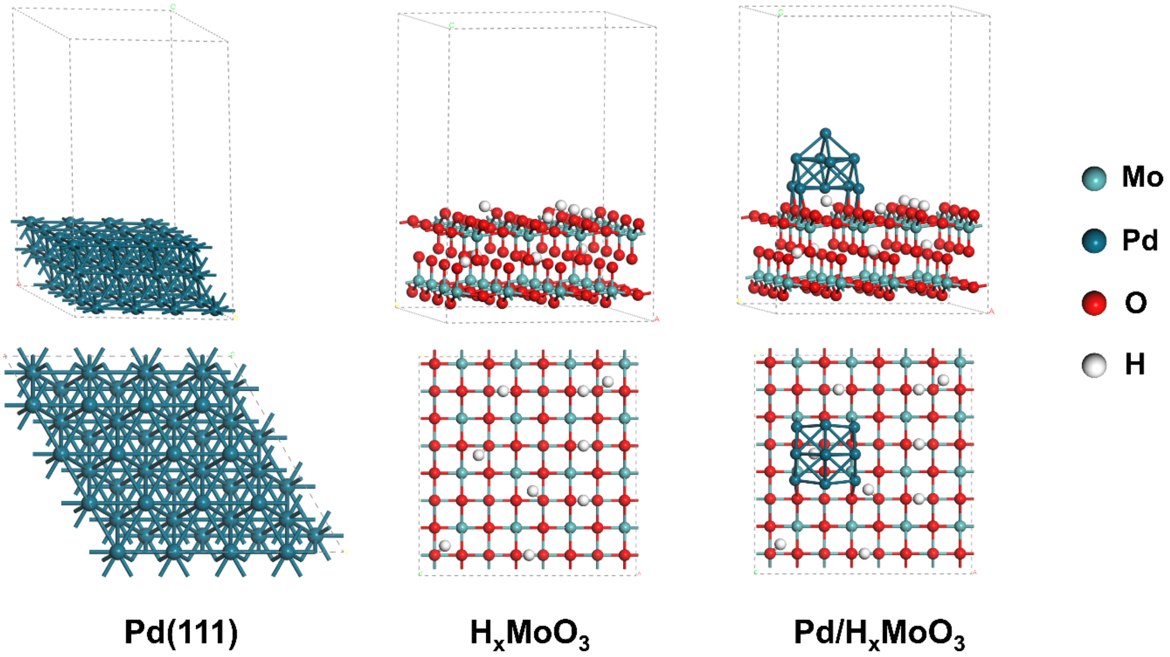

Figure S5. Structural models for Pd (111), H_xMoO_3, and Pd/H_xMoO_3 employed in this work.

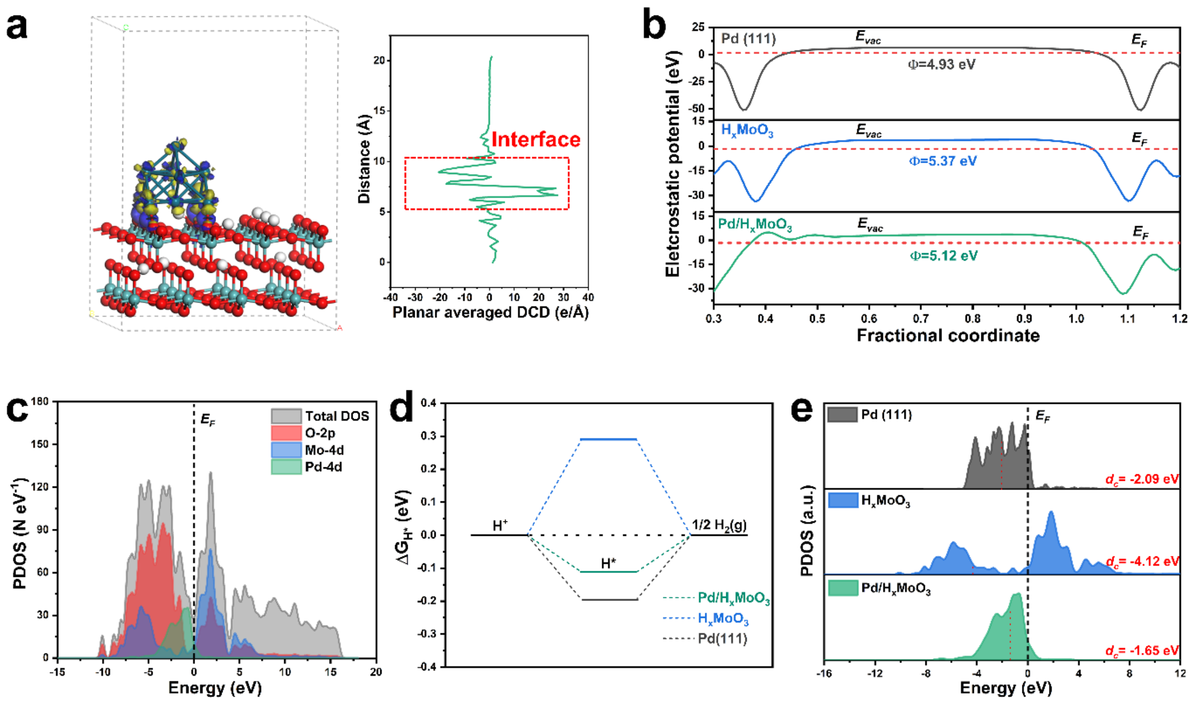

Figure S6. (a, left panel) Charge density differences in the constructed Pd/H_xMoO_3 model (side view). The dark blue and yellow contours represent the regions of electron accumulation and depletion, respectively, where the isosurfaces are set to 0.05 e Å^{-3}. (a, right panel) The plane-averaged differential charge density (DCD) across the interface. (b) Electrostatic potentials for Pd (111), H_xMoO_3, and Pd/H_xMoO_3 models. (c) Projected density of states (PDOS) curves for Pd/H_xMoO_3. (d) Gibbs free energy (ΔG_{H^*}) profiles for the HER and (e) d-PDOS on Pd (111), H_xMoO_3, and Pd/ H_xMoO_3 models (see supporting information for further details).

The Pd/H_xMoO_3 NPs model was constructed according to **Figure S5**. The charge density difference displayed in **Figure S6a** (left panel) shows the accumulation of negative charges at the interface that may facilitate the transfer of LSPR-induced hot charges from H_xMoO_3 to Pd under light irradiation.[1] The corresponding planar average differential charge density (DCD) analysis (**Figure S6a**, right panel) reveals a strong charge redistribution at the interface. This is further demonstrated by the calculated work functions of Pd (111), H_xMoO_3, and Pd/H_xMoO_3 models (**Figure S6b**). Compared with that of the pure Pd, the work function of the Pd/H_xMoO_3 is increased. This occurs

due to the lower Femi energy (E_F) level and a larger work function for H_xMoO_3 relative to Pd. The contact between Pd and H_xMoO_3 induces charge redistribution around the interface until their work functions become equivalent.[2] The projected density of states (PDOS) of various constituent elements in the Pd/H_xMoO_3 model was used to probe the local electronic structures (**Figure S6c**). Mo sites buried at deeper positions far from the E_F possessed an electron-rich character. The Pd sites taking up adjacent positions near E_F assisted in the stable adsorption of the vital intermediate (H*) during the HER process, indicating the high electroactivity. The O 2p orbitals exhibit a broad range of distribution in the valence band and strongly overlap with Pd 4d orbitals, indicative of the efficient p–d coupling over the interfaces that can accelerate electron transfer.[3] For the acidic HER process, the difference in Gibbs free energies (ΔG_{H*}) can be used as a sensitive indicator for the key H* adsorption/desorption step to estimate the HER activity and provide insights into the HER kinetic mechanism.[3] The ΔG_{H*} values for H* adsorption/desorption in Pd, H_xMoO_3, and Pd/H_xMoO_3 models (**Figure S7**) are shown in **Figure S6d.** We observed that Pd/H_xMoO_3 displayed intermediate ΔG_{H*} values (around -0.11 eV), whereas the ΔG_{H*} values for Pd (111) and H_xMoO_3 were lower and higher, respectively. These data suggests that the combination of these materials promote H adsorption, leading to beneficial effects for enhanced HER kinetics.[3] The largely positive ΔG_{H*} value for the H_xMoO_3 sites indicates that H protons cannot efficiently adsorb on the H_xMoO_3 substrate and that the Pd is the active site for the catalysis. Furthermore, we calculated the PDOS of the d orbitals for Pd, H_xMoO_3, and Pd/H_xMoO_3 models and the relative d band center (d_c) for the adsorption of active species (H*). **Figure S6e** shows that the Pd sites in Pd/H_xMoO_3 model shows a more positive d_c than the pure Pd, suggesting enhanced H absorption and HER activity. On the other hand, the d_c of H_xMoO_3 is more negative than that of the Pd-based systems, indicative of the weaker adsorption of H*, which is well consistent with the calculated free energy results. Thus, it is plausible that Pd/H_xMoO_3 can boost the HER activity via the optimization of adsorption/desorption of H* species

(both under dark and light conditions) and the facilitation of LSPR-excited charge carriers transfer (from H_xMoO_3) to Pd NPs under light excitation.

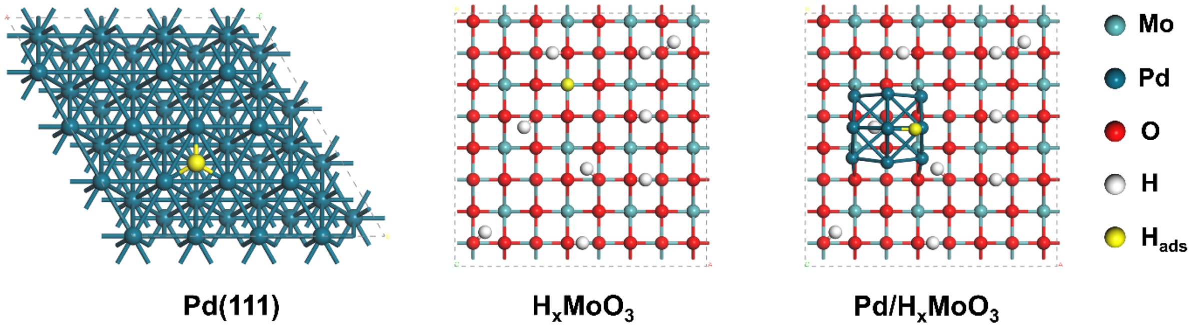

Figure S7. Possible hydrogen-adsorption sites over Pd (111), H_xMoO_3, and Pd/H_xMoO_3 surfaces.

Table S1. Conversion, selectivity, and photo-enhancement for the hydrogenation of phenylacetylene to styrene as a function of irradiation wavelength.

Entry	Catalyst	Light source (nm)	Conversion (%)	Selectivity (%)	Photo-enhancement (%)	TOF (h^{-1})
1	Pd/H_xMoO_3	Dark	29	96	-	1239
2	Pd/H_xMoO_3	525	35	96	21	1496
3	Pd/H_xMoO_3	740	38	95	31	1624
4	Pd/H_xMoO_3	640	39	95	34	1667
5	Pd/H_xMoO_3	427	41	96	41	1752
6	Pd/H_xMoO_3	427 & 640	62	94	114	2650

[a]The reactions were conducted under H_2 atmosphere (1 bar), with 10 mL of isopropanol, 0.5 mmol of substrate and 1.5 mg of catalyst. The reaction time was 1 h.

A

B

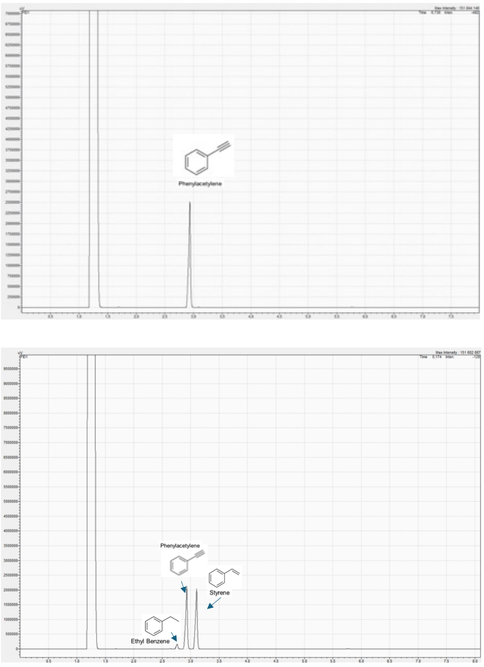

Figure S8. Gas chromatograms obtained from the reaction mixture before (A) and after (B) the reaction (entry 6, Table S1).

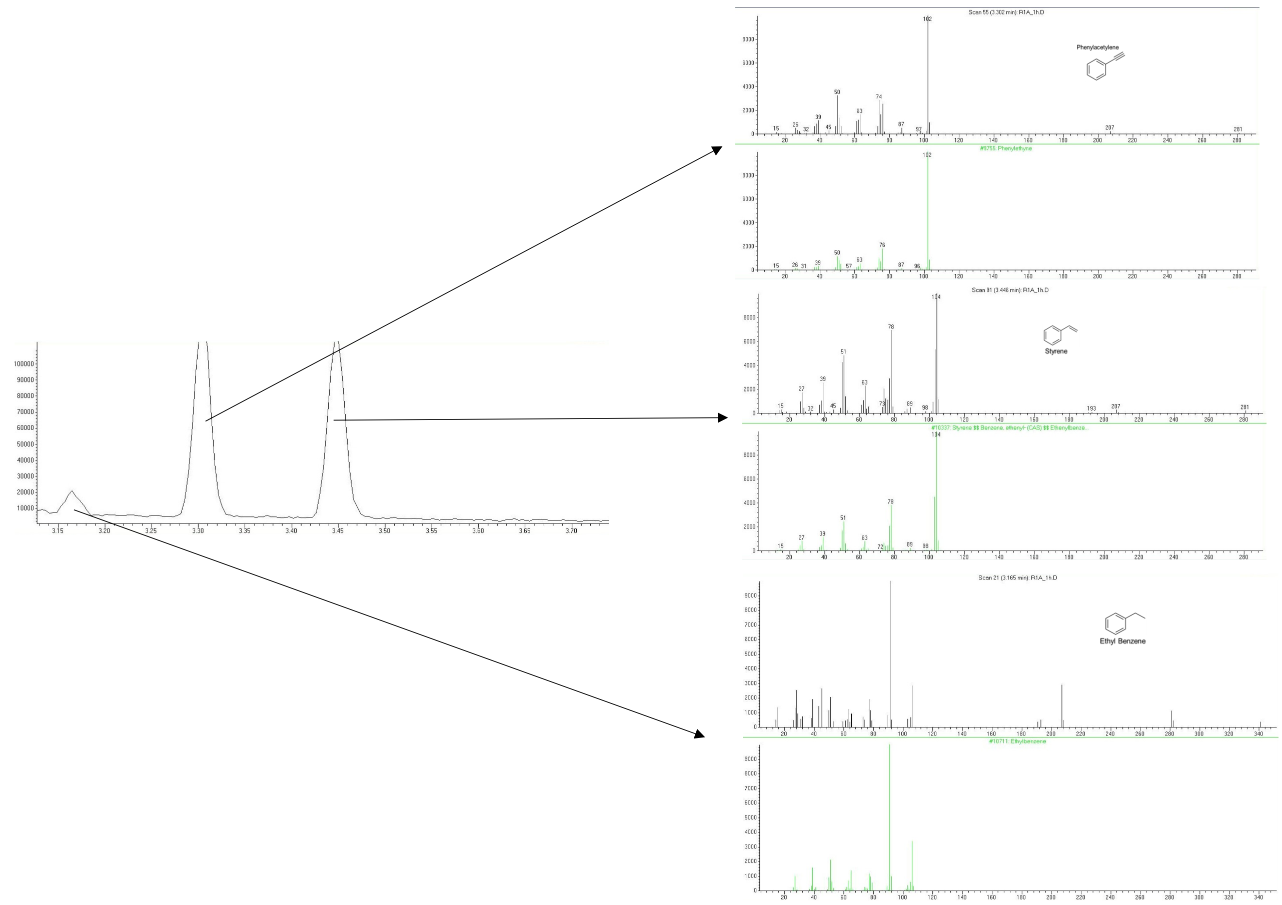

Figure S9. GC-MS analysis of the reaction mixture after the phenylacetylene hydrogenation (entry 6, Table S1). Standard MS spectra for phenylacetylene, styrene, and ethylbenzene are shown in green.

Table S2. Substrate scope investigation of the catalytic hydrogenation of alkynes by Pd/H_xMoO_3 under visible light irradiation under 427 and 640 nm (double light excitation conditions)

Entry	Substrate	Conversion (%)	Selectivity (%)
1	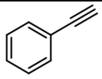	62	94
2	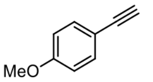	61	91
3	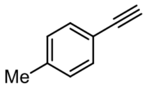	77	92
4	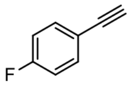	100	0

[a]The reactions were conducted under H_2 atmosphere (1 bar), with 10 mL of isopropanol, 0.5 mmol of substrate and 1.5 mg of catalyst. The reaction time was 1 h.

Table S3. Comparison of Pd/H_xMoO_3 and other reported catalysts for hydrogenation of phenylacetylene.

	T (°C)	P (bar)	t (h)	Molar ratio Pd/PA	Conv. (%)	Sel. (%)	TOF (h^{-1})	Ref.
Pd/H_xMoO_3	40	1	1	$2.3x10^{-4}$	62	94	2650	This work
Pd NCs/C	110	10	1.5	$2.8x10^{-3}$	100	47	155	4
$Pd_{4.5}Se$ NCs/C	110	10	1.5	$2.8x10^{-3}$	100	99	351	4
Pd/NHPC-DETA	35	1	-	$3.7x10^{-4}$	99	95	2872	5
SAC Pd_1/Ni@G	30	2	1	$1.3x10^{-4}$	100	93	7074	6
MS Pd-Ru@ZIF-8	100	1	2	$5.5x10^{-4}$	98	96	2188	7
SAC 0.2Pd-TiO_2	25	10	0.5	$2.3x10^{-1}$	99	91	8596	8
SAC PdIn/$MgAl_2O_4$	25	1	3.5	$3.8x10^{-3}$	92	97	70	9
nano-Pd/α-Al_2O_3	50	10	0.5	$1.9x10^{-4}$	100	50	11.8 (s^{-1})	10
CeO_2/Pd@MIL-53	25	1	1	$1.0x10^{-3}$	100	96	16 (s^{-1})	11
PdZn+0.25Pb	25	1	25	$5.6x10^{-3}$	99	94	450 (min^{-1})	12

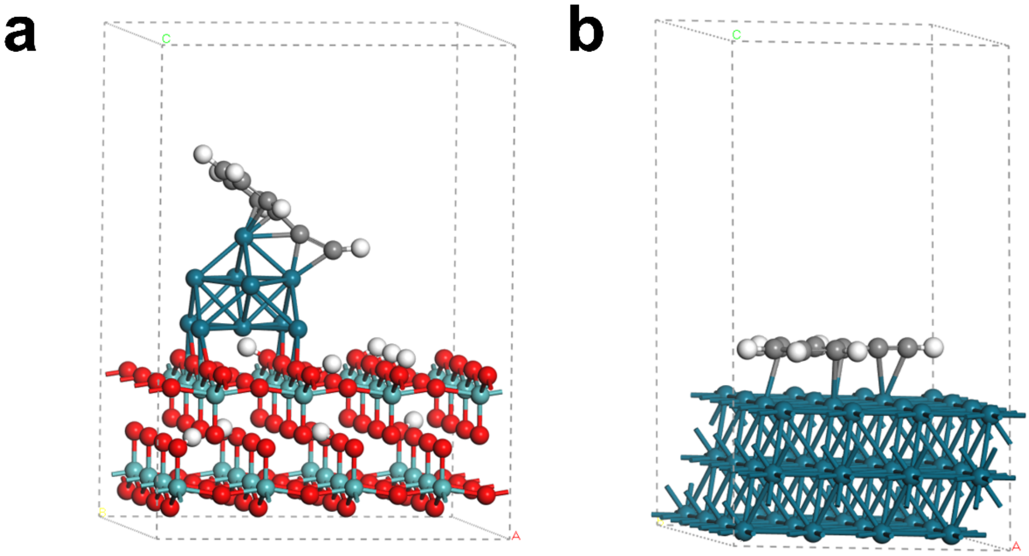

Figure S10. The optimized structure for phenylacetylene adsorption over (a) Pd/H_xMoO_3 and (b) Pd (111) models.

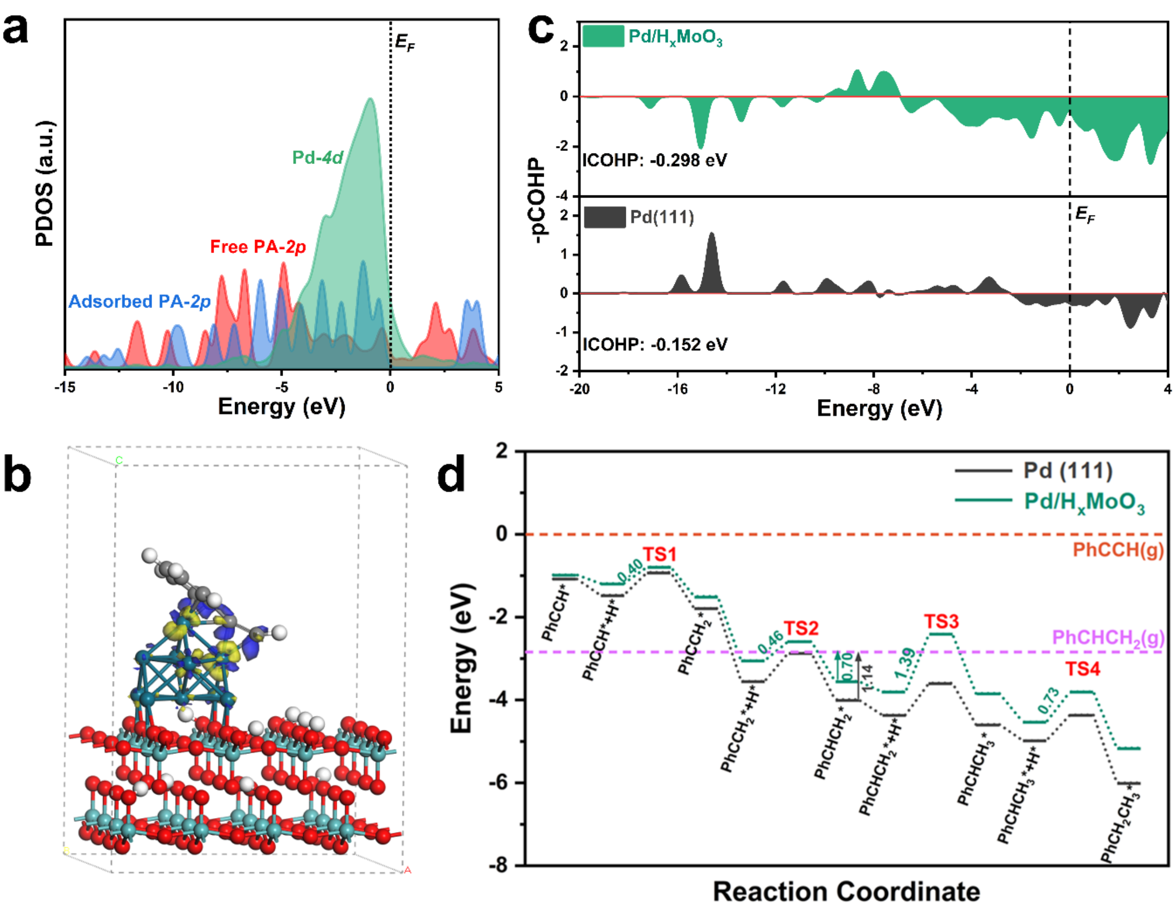

Figure S11. (a) PDOS for a free (red) and adsorbed (blue) phenylacetylene molecule (2p levels) on the surface of Pd/H_xMoO_3 model. (b) Charge density difference analysis in the constructed Pd/H_xMoO_3 model containing a phenylacetylene molecule adsorbed at the Pd cluster site. The dark blue and yellow contours represent the regions of electron accumulation and depletion, where the isosurfaces are set to 0.05 e Å^{-3}. (c) COHP bonding analysis of Pd-C interactions (Pd in the surface site and C in adsorbed phenylacetylene). (d) Free energy profiles for the phenylacetylene hydrogenation reaction on Pd (111) and Pd/H_xMoO_3 models.

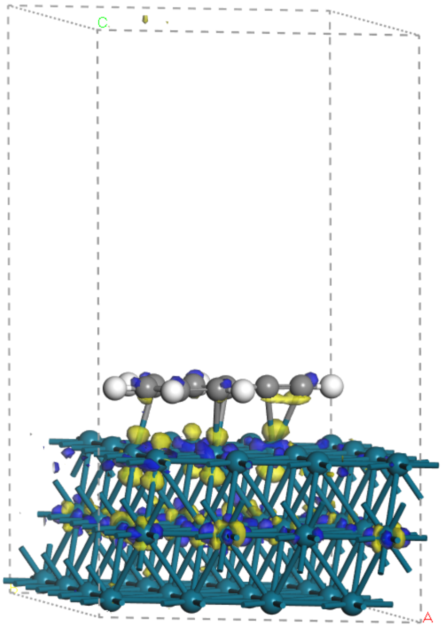

Figure S12. Charge density difference analysis for a phenylacetylene adsorbed in the constructed Pd (111) model The dark blue and yellow contours represent the regions of electron accumulation and depletion, where the isosurfaces are set to 0.05 e Å^{-3}.

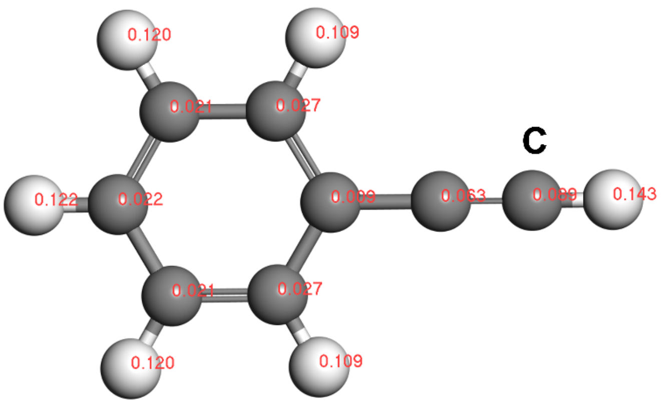

Figure S13. Structural model of the phenylacetylene molecule with the Fukui charge distribution. Due to the large Fukui charge of the labeled C atom, we choose this corresponding Pd-C bond (after adsorption) for the further COHP analysis (**Figure S11C**).

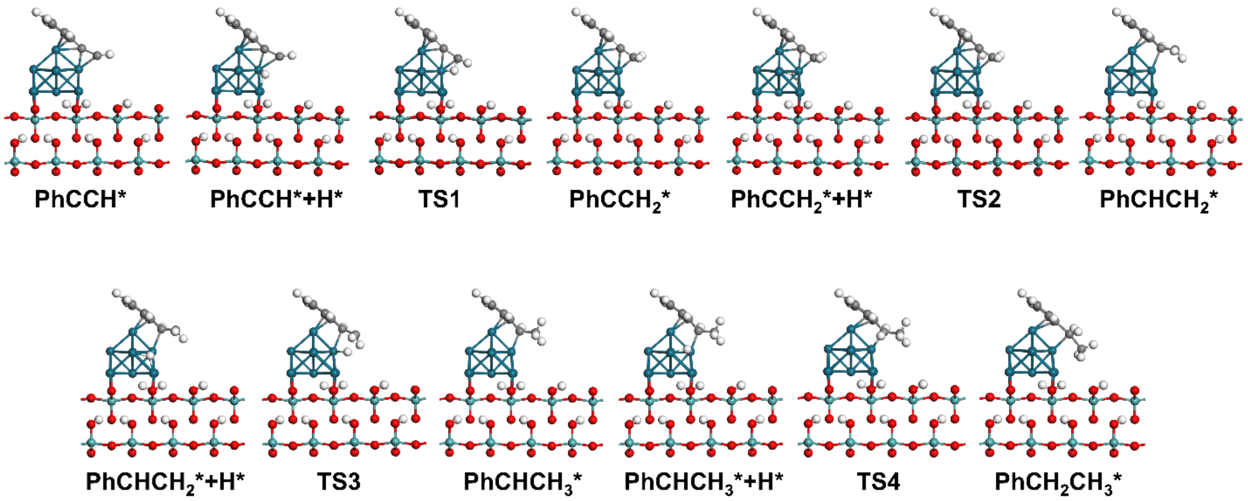

Figure S14. The corresponding configurations for the phenylacetylene hydrogenation reaction on the Pd/H_xMoO_3 model.

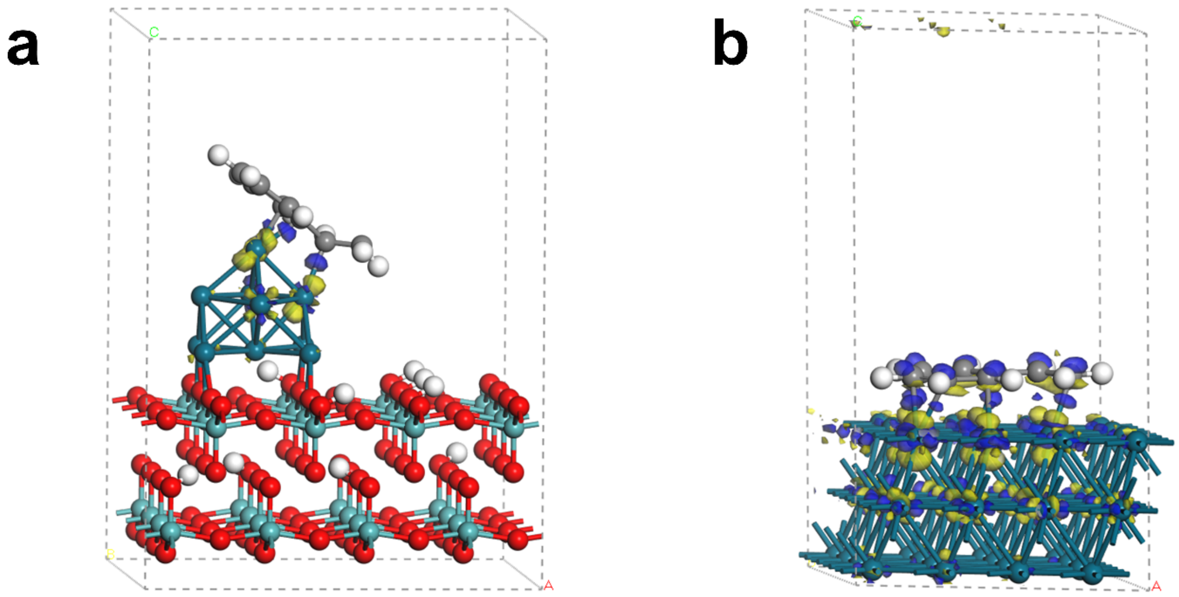

Figure S15. Charge density difference analysis for a styrene molecule adsorbed in the constructed (a) Pd/H_xMoO_3 and (b) Pd (111) models. The dark blue and yellow contours represent the regions of electron accumulation and depletion, where the isosurfaces are set to 0.05 e Å^{-3}.

References

(1) Bai, H.; Hei Lam, S.; Yang, J.; Cheng, X.; Li, S.; Jiang, R.; Shao, L.; Wang, J.; Bai, H. Y.; Lam, S. H.; Yang, J. H.; Cheng, X. Z.; Li, S. S.; Wang, J. F.; Jiang, R. B.; Shao, L. A Schottky-Barrier-Free Plasmonic Semiconductor Photocatalyst for Nitrogen Fixation in a "One-Stone-Two-Birds" Manner. *Advanced Materials* **2022**, *34* (2), 2104226. https://doi.org/10.1002/ADMA.202104226.

(2) Wang, Y.; Hu, J.; Ge, T.; Chen, F.; Lu, Y.; Chen, R.; Zhang, H.; Ye, B.; Wang, S.; Zhang, Y.; Ma, T.; Huang, H. Gradient Cationic Vacancies Enabling Inner-To-Outer Tandem Homojunctions: Strong Local Internal Electric Field and Reformed Basic Sites Boosting CO2 Photoreduction. *Advanced Materials* **2023**, *35* (31), 2302538. https://doi.org/10.1002/ADMA.202302538.

(3) Li, L.; Bu, L.; Huang, B.; Wang, P.; Shen, C.; Bai, S.; Chan, T. S.; Shao, Q.; Hu, Z.; Huang, X. Compensating Electronic Effect Enables Fast Site-to-Site Electron Transfer over Ultrathin RuMn Nanosheet Branches toward Highly Electroactive and Stable Water Splitting. *Advanced Materials* **2021**, *33* (51). https://doi.org/10.1002/ADMA.202105308.

(4) Wang, M.; Liang, L.; Liu, X.; Sun, Q.; Guo, M.; Bai, S.; Xu, Y. Selective Semi-Hydrogenation of Alkynes on Palladium-Selenium Nanocrystals. *J Catal* **2023**, *418*, 247–255. https://doi.org/10.1016/j.jcat.2023.01.024.

(5) Luo, Q.; Wang, Z.; Chen, Y.; Mao, S.; Wu, K.; Zhang, K.; Li, Q.; Lv, G.; Huang, G.; Li, H.; Wang, Y. Dynamic Modification of Palladium Catalysts with Chain Alkylamines for the Selective Hydrogenation of Alkynes. *ACS Appl Mater Interfaces* **2021**, *13* (27), 31775–31784. https://doi.org/10.1021/acsami.1c09682.

(6) Zhao, L.; Qin, X.; Zhang, X.; Cai, X.; Huang, F.; Jia, Z.; Diao, J.; Xiao, D.; Jiang, Z.; Lu, R.; Wang, N.; Liu, H.; Ma, D. A Magnetically Separable Pd Single-Atom Catalyst for Efficient Selective

Hydrogenation of Phenylacetylene. *Advanced Materials* **2022**, *34* (20), 2110455. https://doi.org/10.1002/adma.202110455.

(7) Li, Z.; Hu, M.; Liu, J.; Wang, W.; Li, Y.; Fan, W.; Gong, Y.; Yao, J.; Wang, P.; He, M.; Li, Y. Mesoporous Silica Stabilized MOF Nanoreactor for Highly Selective Semi-Hydrogenation of Phenylacetylene via Synergistic Effect of Pd and Ru Single Site. *Nano Res* **2022**, *15* (3), 1983–1992. https://doi.org/10.1007/s12274-021-3849-2.

(8) Yang, F.; Ding, S.; Song, H.; Yan, N. Single-Atom Pd Dispersed on Nanoscale Anatase TiO2 for the Selective Hydrogenation of Phenylacetylene. *Sci China Mater* **2020**, *63* (6), 982–992. https://doi.org/10.1007/s40843-020-1271-x.

(9) Feng, Q.; Zhao, S.; Wang, Y.; Dong, J.; Chen, W.; He, D.; Wang, D.; Yang, J.; Zhu, Y.; Zhu, H.; Gu, L.; Li, Z.; Liu, Y.; Yu, R.; Li, J.; Li, Y. Isolated Single-Atom Pd Sites in Intermetallic Nanostructures: High Catalytic Selectivity for Semihydrogenation of Alkynes. *J Am Chem Soc* **2017**, *139* (21), 7294–7301. https://doi.org/10.1021/jacs.7b01471.

(10) Hu, J.; Zhou, Z.; Zhang, R.; Li, L.; Cheng, Z. Selective Hydrogenation of Phenylacetylene over a Nano-Pd/α-Al2O3 Catalyst. *J Mol Catal A Chem* **2014**, *381*, 61–69. https://doi.org/10.1016/j.molcata.2013.10.008.

(11) Li, X.; Song, L.; Gao, D.; Kang, B.; Zhao, H.; Li, C.; Hu, X.; Chen, G. Tandem Catalysis of Ammonia Borane Dehydrogenation and Phenylacetylene Hydrogenation Catalyzed by CeO 2 Nanotube/Pd@MIL-53(Al). *Chemistry – A European Journal* **2020**, *26* (19), 4419–4424. https://doi.org/10.1002/chem.202000085.

(12) Miyazaki, M.; Furukawa, S.; Takayama, T.; Yamazoe, S.; Komatsu, T. Surface Modification of PdZn Nanoparticles via Galvanic Replacement for the Selective Hydrogenation of Terminal Alkynes. *ACS Appl Nano Mater* **2019**, *2* (5), 3307–3314. https://doi.org/10.1021/acsanm.9b00761.
